# Synthesis of metal-fluoride nanoparticles supported on thermally reduced graphite oxide

**DOI:** 10.3762/bjnano.8.247

**Published:** 2017-11-22

**Authors:** Alexa Schmitz, Kai Schütte, Vesko Ilievski, Juri Barthel, Laura Burk, Rolf Mülhaupt, Junpei Yue, Bernd Smarsly, Christoph Janiak

**Affiliations:** 1 Institut für Anorganische Chemie und Strukturchemie, Heinrich-Heine-Universität Düsseldorf, 40204 Düsseldorf, Germany; 2 Gemeinschaftslabor für Elektronenmikroskopie RWTH-Aachen, Ernst Ruska-Centrum für Mikroskopie und Spektroskopie mit Elektronen, D-52425 Jülich, Germany; 3 Freiburg Materials Research Center and Institute for Macromolecular Chemistry, Albert-Ludwigs-University Freiburg, 79104 Freiburg, Germany; 4 Physikalisch-Chemisches Institut, Justus-Liebig-Universität Gießen, 35392 Gießen, Germany

**Keywords:** ionic liquids, material synthesis, metal-fluoride nanoparticles, microwave irradiation, thermally reduced graphite oxide

## Abstract

Metal-fluoride nanoparticles, (MF*_x_*-NPs) with M = Fe, Co, Pr, Eu, supported on different types of thermally reduced graphite oxide (TRGO) were obtained by microwave-assisted thermal decomposition of transition-metal amidinates, (M{MeC[N(iPr)]_2_}*_n_*) or [M(AMD)*_n_*] with M = Fe(II), Co(II), Pr(III), and tris(2,2,6,6-tetramethyl-3,5-heptanedionato)europium, Eu(dpm)_3_, in the presence of TRGO in the ionic liquid (IL) 1-butyl-3-methylimidazolium tetrafluoroborate ([BMIm][BF_4_]). The crystalline phases of the metal fluorides synthesized in [BMIm][BF_4_] were identified by powder X-ray diffraction (PXRD) to be MF_2_ for M = Fe, Co and MF_3_ for M = Eu, Pr. The diameters and size distributions of MF*_x_*@TRGO were from (6 ± 2) to (102 ± 41) nm. Energy-dispersive X-ray spectroscopy (EDX) and X-ray photoelectron spectroscopy (XPS) were used for further characterization of the MF*_x_*-NPs. Electrochemical investigations of the FeF_2_-NPs@TRGO as cathode material for lithium-ion batteries were evaluated by galvanostatic charge/discharge profiles. The results indicate that the FeF_2_-NPs@TRGO as cathode material can present a specific capacity of 500 mAh/g at a current density of 50 mA/g, including a significant interfacial charge storage contribution. The obtained nanomaterials show a good rate capacity as well (220 mAh/g and 130 mAh/g) at a current density of 200 and 500 mA/g, respectively.

## Introduction

Graphene is the parent compound of all graphitic carbon forms and a form of nanocarbon [1]. It has a large specific surface, is electrically and thermally conductive and has a high mechanical resistance [2]. The International Union of Pure and Applied Chemistry (IUPAC) defines graphene as an isolated two-dimensional monolayer of sp^2^-hybridized carbon atoms [3], extended in a honeycomb-type structure that consist of six-membered rings [3]. Functionalized graphene is obtained from graphite by graphite oxidation followed by thermal reduction. During the thermal reduction of graphite oxide by flash pyrolysis, the decomposition of epoxy, carbonyl and carboxyl groups accounts for a build-up of pressure that exfoliates functionalized graphene [4].

In 1958, Hummers and Offeman reported on a ”graphene” synthesis by oxidation of graphite with sodium nitrate, potassium permanganate and sulfuric acid followed by thermal reduction through rapid heating under nitrogen to 300–1000 °C [5], yielding thermally reduced graphite oxide (TRGO) as a graphene-type material (Scheme S1, Supporting Information File 1) [6]. The thermal reduction results in the loss of most of the oxygen functionalities on the surface. This can be controlled by varying the reduction temperature, yielding different types of TRGO characterized by decreasing oxygen functionality with increasing temperature [7]. Due to its remaining oxygen functionalities and its porosity TRGO is an attractive carrier material for the immobilization of very small nanoparticles [8–12].

In 2009, the first nanoparticles@TRGO were synthesized by heating graphite oxide with Pt, Ru or Pd complexes under a nitrogen atmosphere [13]. Alternatively, salts of palladium and other metals are readily immobilized on graphene oxide by means of cation exchange with carboxylic acid groups, followed by thermal reduction to produce metal nanoparticles supported on functionalized graphene. Such palladium nanoparticles supported on graphene were used as highly active catalysts for the Suzuki–Miyaura coupling reaction [14]. In 2011, metal carbonyls in dispersion with TRGO and ionic liquid (IL) were exposed to short low-energy microwave irradiation. The resulting Ru@TRGO and Rh@TRGO particles had high catalytic hydrogenation activity [12]. Metallic nanoparticles on graphene have important technical applications [15–22]. They can be used as composite materials [23–24], in chemical sensors [25], electrodes for fuel cells [26–28], for catalysis [29–32] or for hydrogen storage [33].

Because of their high ionic charge, polarity and dielectric constant, ILs are an ideal media for microwave reactions and for the stabilization of M-NPs [34–37]. Soft wet-chemical synthesis in organic solvents from metal-organic complexes is an essential method to obtain metal or metal alloy nanoparticles [38–50].

The synthesis of inorganic nanomaterials is thoroughly investigated but still requires well-established, simple protocols with inexpensive and non-toxic chemicals for many of the important inorganic nanoparticles [51–52]. Metal-fluoride nanoparticles, MF*_x_*-NPs are important in materials science and modern chemistry [53–54]. Nanoscale main-group metal fluorides can be obtained from a fluorolytic sol–gel route by the reaction of the metal alkoxide or acetate with anhydrous HF in a suitable organic solvent. Strong Lewis-acidic main-group metal fluorides such as AlF_3_ or MgF_2_ represent a new class of heterogeneous nanocatalysts [55–56]. Transition-metal-fluoride nanoparticles are applied, for example, as cathode materials in lithium-ion batteries for vehicles and other mobile devices [57]. In this field, the modification of lithium–transition-metal electrodes is a very important issue to improve the performance of lithium-ion batteries [58–61].

**Scheme 1:**
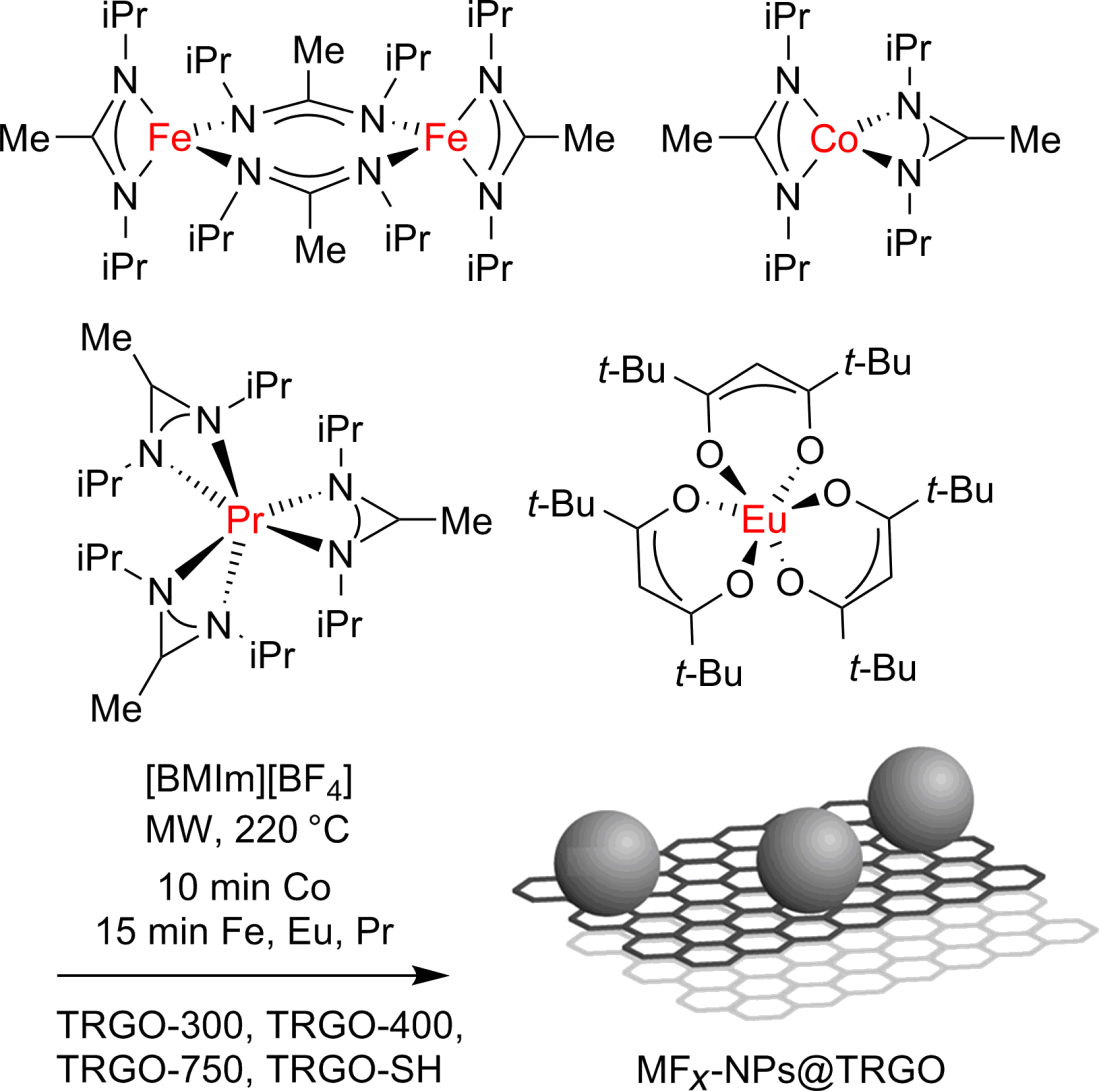
Synthesis scheme of MF*_x_*@TRGO from [M(AMD)*_n_*] and [Eu(dpm)_3_] by microwave (MW)-assisted thermal decomposition on thermally reduced graphite oxide (TRGO) in the ionic liquid [BMIm][BF_4_].

Herein, we report on the utilization of metal amidinates (M{MeC[N(iPr)]_2_}*_n_* or M(AMD)*_n_*) of iron, cobalt and praseodymium and of tris(2,2,6,6-tetramethyl-3,5-heptanedionato)europium, Eu(dpm)_3_ as precursors with different types of TRGO for the synthesis of nanocomposite materials in ionic liquids (ILs) to yield selectively phase-pure metal-fluoride nanoparticles (MF*_x_*-NPs) supported on the TRGO as stable colloids (Scheme 1). The used TRGO starting materials differed in the temperatures at which they were reduced (300, 400 or 750 °C) and in the presence of sulfur functionalities.

## Results and Discussion

Transition-metal amidinates [M(AMD)*_n_*; M = Fe(II), Co(II), Pr(III)] as well as Eu(dpm)_3_ were dissolved or suspended under nitrogen atmosphere in the dried and deoxygenated ionic liquid together with the selected type of thermally reduced graphene oxide (TRGO). Complete decomposition by microwave irradiation of the precursors in IL was achieved after only 10 min for Co(II) and 15 min for Fe(II), Eu(III) and Pr(III) using a low power of 50 W to give a temperature of 220 °C in the reaction mixture (Scheme 1). Black dispersions of nanocomposite materials were reproducibly obtained by repeated decompositions of the precursors/TRGO.

Different types of TRGO were employed for the deposition of the metal-fluoride nanoparticles. The number value of the suffix at TRGO specifies the temperature (in degree Celsius) that was used to reduce the graphite oxide to TRGO (Scheme S1 and Scheme S2 in Supporting Information File 1). Thiol-functionalized TRGO-SH [62] was additionally used to support metal-fluoride nanoparticles.

**Figure 1:**
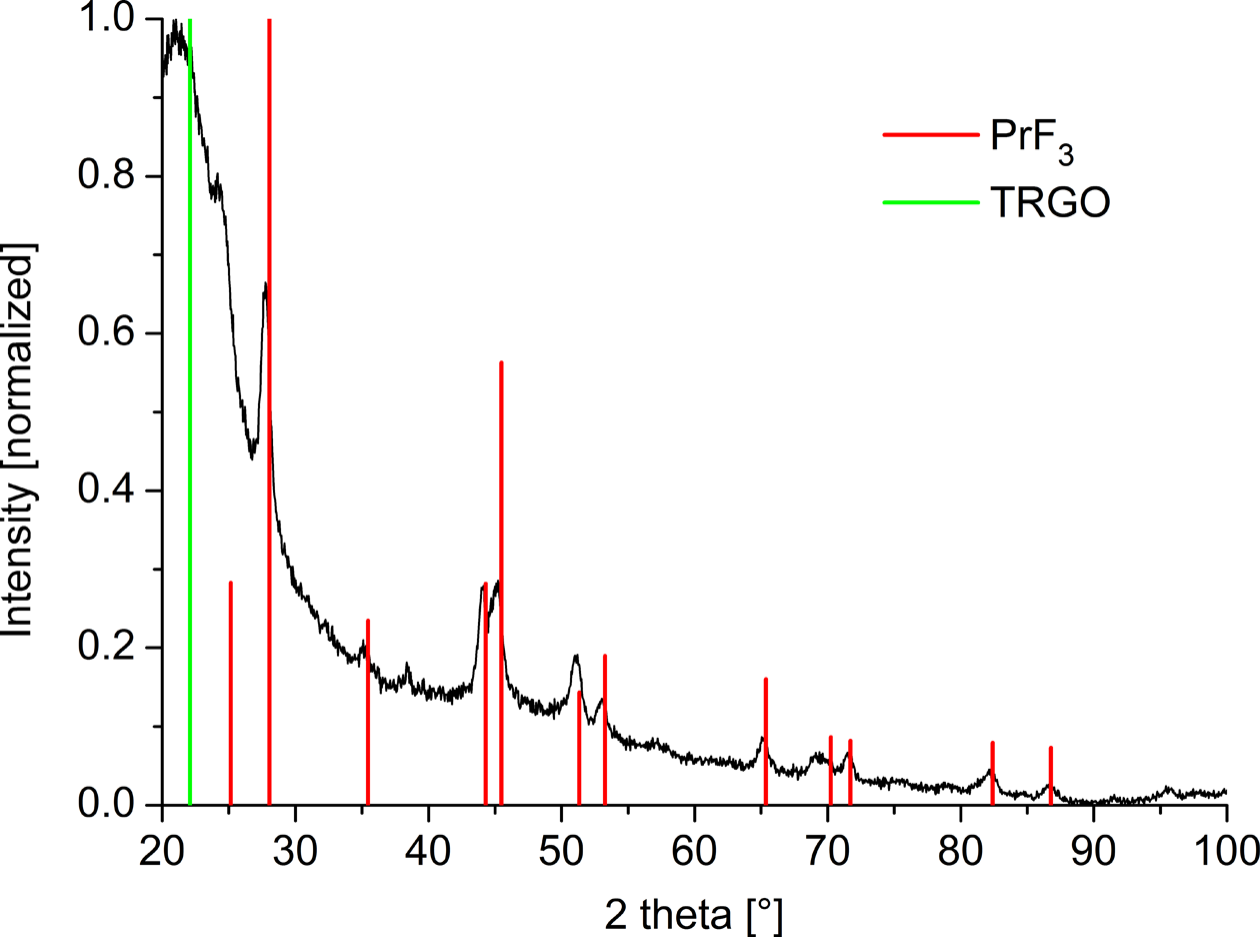
Example PXRD of 0.5 wt % PrF_3_@TRGO-SH in [BMIm][BF_4_] synthesized from [Pr(AMD)_3_]. PrF_3_ reference reflections in red from COD 1010984. For the diffractogram with indexed reflections see Figure S18 in Supporting Information File 1. The PXRDs for the other samples are given in Figures S4–S19 in Supporting Information File 1.

The morphology, crystalline phase (MF*_x_*-NPs), size and size dispersion of the nanoparticles was analyzed by powder X-ray diffraction (PXRD) and transmission electron microscopy (TEM). X-ray photo electron spectroscopy (XPS) was used to determine the metal/fluoride ratio, as well as the metal oxidation state. The crystalline phase analysis was based on positively matching the experimental powder X-ray diffractograms (PXRDs) to metal-fluoride structures deposited in the crystallographic open database (COD) (Figures S4–S19, Supporting Information File 1). For example, for PrF_3_ the PXRD matches the hexagonal close-packed (hcp) structure of praseodymium metal trifluoride with space group *P*6_3_/*mcm* (Figure 1).

The formation of metal fluorides instead of metal nanoparticles in the IL [BMIm][BF_4_] must be rationalized from the fluoride content of the IL tetrafluoroborate anion. It is known that the [BF_4_]^−^ anion hydrolyzes or decomposes to fluoride, F^−^, in the presence of small amounts of residual water in the IL, which is very difficult to remove from hydrophilic [BMIm][BF_4_] [63]. Water is used in IL synthesis during the washing process following the anion exchange from typically chloride to tetrafluoroborate. Anion analyses by ion chromatography of the purified ILs yielded fluoride contents of 0.1 to 0.4 wt % for [BMIm][BF_4_] ILs [64]. Heating the ILs for the amidinate decomposition may lead to further hydrolysis of [BF_4_]^−^ and fluoride formation with the residual water. Fluoride ions can then lead to the formation of metal fluorides [65]. Alternatively, reactive metal atoms or metal clusters may also abstract fluoride from [BF_4_]^−^ anions.

ILs are already recognized as solvents and as reactants. In the synthesis of nanoparticles of the fluoridosilicates A_2_SiF_6_ (A = Li, Na, K, Rb, Cs) the IL [BMIm][PF_6_] was used both as solvent and fluoride source in an ionothermally assisted microwave synthesis [66]. Decomposition of transition-metal amidinates in [BMIm][BF_4_] yielded metal fluoride nanoparticles for Mn, Fe and Co [63]. The reaction of metal acetate (hydrate) precursors in ethylene glycol and an excess of [BMIm][BF_4_] gave fluoride nanoparticles [67]. Mesoporous carbon/iron carbide hybrids were synthesized using mesoporous silica as template and the ionic liquid [BMIm][FeCl_4_] as carbon and iron source [68]. CuCl nanoplatelets were obtained from mixtures of a Cu-containing ionic liquid crystal and 6-O-palmitoyl ascorbic acid [69].

**Figure 2:**
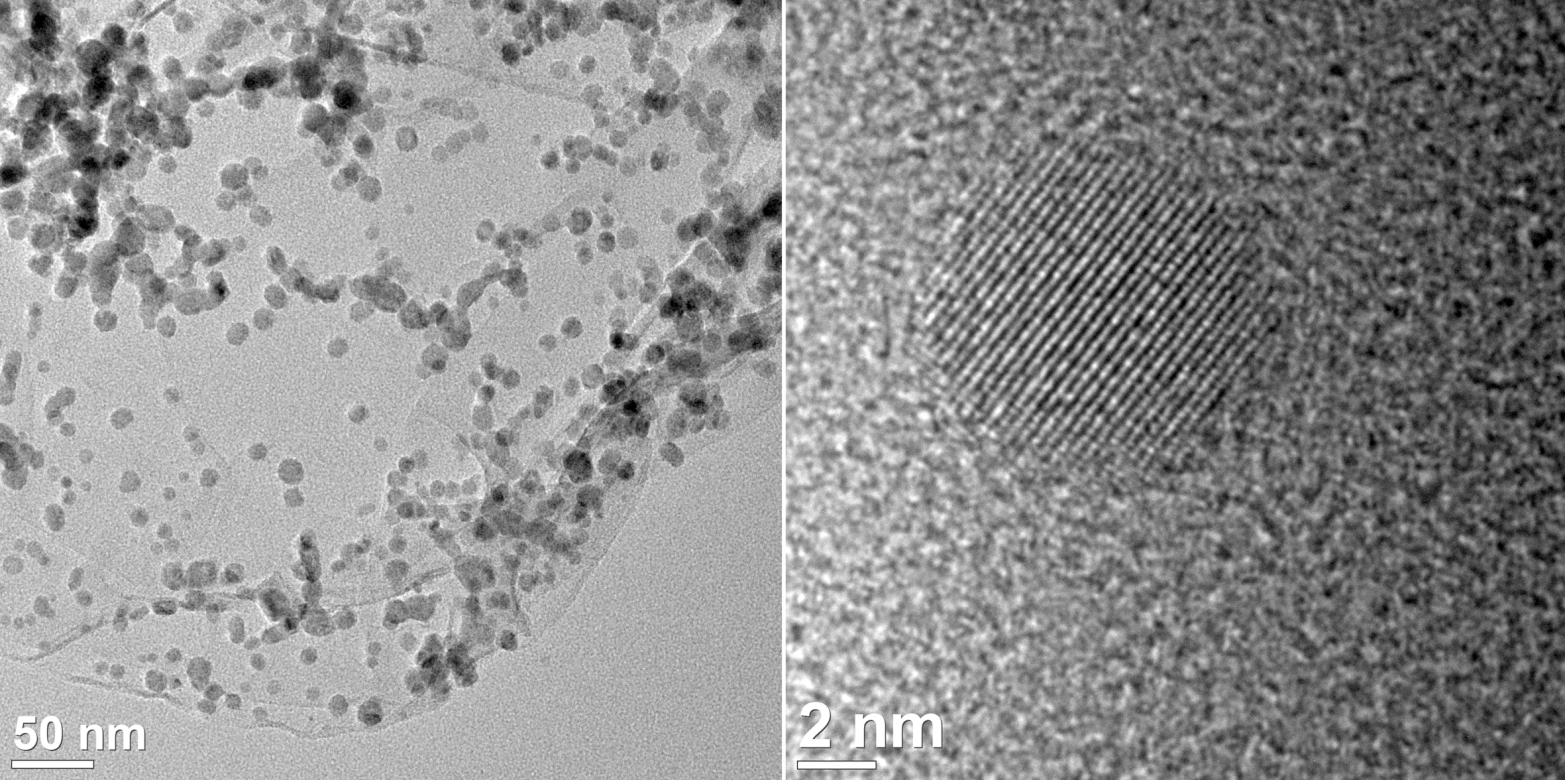
TEM images of PrF_3_@TRGO-400 dispersions from [Pr(AMD)_3_] in [BMIm][BF_4_].

Analysis of the MF*_x_*@TRGO nanocomposite materials by (high-resolution) transmission electron microscopy ((HR-)TEM) (Figure 1, Figures S4–S19 in Supporting Information File 1) indicated the formation of MF*_x_* nanoparticles with typical diameters between 4 and 30 nm supported on the TRGO. The sizes and size dispersions of the metal-fluoride nanoparticles are summarized in Table 1. The diameters of the MF*_x_* nanoparticles were derived from evaluation of as many reflections as possible in the powder X-ray diffractograms by using the Scherrer equation. Further, sizes and size dispersion were obtained from measuring at least 50 particles in the TEM images (Figure 2 and Supporting Information File 1). High-resolution TEM images frequently showed interference patterns (lattice planes), which is an indication of crystallinity. For the iron difluoride nanoparticles nanorods were obtained besides nanoparticles (Figures S4, S8, S12 and S16 in Supporting Information File 1). For praseodymium trifluoride, spherical crystalline nanoparticles were found with clear interference patterns within the particles (Figure 2 and Figure S6, S10, S14, S18 in Supporting Information File 1). Eu(dpm)_3_ gave crystalline facetted particles that laid partly next to the TRGO (Figures S7, S11 and S19 in Supporting Information File 1). After the reaction of cobalt amidinate on TRGO-300 to TRGO-750 rather aggregated metal assemblies were obtained under the used reaction conditions so that individual particles were difficult to discern and no clear sizes could be derived (Figures S9 and S13 in Supporting Information File 1). TRGO-SH was derived from TRGO-400 by reaction with lithium diisopropylamide (LDA) and propylene sulfide. Subsequently, the TRGO-SH carries sulfur functionalities on the surface that were intended to increase the interactions with the nanoparticles (see Scheme S2, Supporting Information File 1) [7]. Also, from cobalt amidinate individual CoF_2_ nanoparticles could be deposited on TRGO-SH, showing interference patterns within the particles (Figure S17, Supporting Information File 1).

**Table 1:** Determined sizes of MF*_x_*-NPs in MF*_x_*@TRGO samples.^a^

precursor	identified phase of MF*_x_*-NPs^b^ on TRGO	NP diameter from PXRD [nm]^c^	particle diameter from TEM [nm]^d,e^	particle diameter without TRGO from TEM [nm]^d^

TRGO-300				

Fe(AMD)_2_	FeF_2_	8–30	26 ± 7 102 ± 41^f^	65 ± 18^g^
Co(AMD)_2_	—^h^	16–31	–^h^	43 ± 11^g^
Pr(AMD)_3_	PrF_3_	9–17	15 ± 4	11 ± 6^g^
Eu(dpm)_3_	EuF_3_	15–21	14 ± 6	21 ± 7^i^

TRGO-400				

Fe(AMD)_2_	FeF_2_	9–20	30 ± 10	65 ± 18^g^
Co(AMD)_2_	—^h^	16–31	—^h^	43 ± 11^g^
Pr(AMD)_3_	PrF_3_	10–14	10 ± 3	11 ± 6^g^
Eu(dpm)_3_	EuF_3_	13–21	14 ± 4	21 ± 7^i^

TRGO-750				

Fe(AMD)_2_	FeF_2_	10–26	6 ± 2	65 ± 18^g^
Co(AMD)_2_	—^h^	21–38	—^h^	43 ± 11^g^
Pr(AMD)_3_	PrF_3_	8–16	17 ± 4	11 ± 6^g^
Eu(dpm)_3_	EuF_3_	14–22	18 ± 4	21 ± 7^i^

TRGO-SH				

Fe(AMD)_2_	FeF_2_	16-28	6 ± 2	65 ± 18^g^
Co(AMD)_2_	CoF_2_	—^j^	9 ± 2	43 ± 11^g^
Pr(AMD)_3_	PrF_3_	14-21	6 ± 2	11 ± 6^g^
Eu(dpm)_3_	EuF_3_	13-23	15 ± 5	21 ± 7^i^

^a^0.5 wt % MF*_x_*-NP/[BMIm][BF_4_] dispersions obtained by microwave-assisted heating for 10 min for Co, 15 min for Fe, Pr and Eu precursors; ^b^the phases of the nanoparticles were identified from PXRD; ^c^diameter calculated from Scherrer equation, Scherrer factor = 1; anisotropic defects were not considered; a range is given for diameter values derived from different reflections; ^d^average diameter and standard deviation σ; ^e^see Experimental section for TEM measurement conditions; at least 50 particles were used for the analysis; ^f^width and length of the rods; ^g^data from [63]; ^h^no separated nanoparticles; ^i^data from [76]; ^j^no reflections in PXRD.

From various mineral studies the substitution of F^−^ with OH^−^ is well known [70–75]. Hence, it is possible that the fluoride ions in the metal fluoride nanoparticles can be partially substituted by hydroxide ions from traces of residual water. At the level of analysis that is possible with the MF*_x_* nanoparticles we cannot, however, quantify any oxygen content in the metal-fluoride nanoparticles. In comparison to earlier works on the formation of metal-fluoride nanoparticles in [BMIm][BF_4_] the MF*_x_* particles had a different size when deposited on TRGO, however, with no clear trend concerning an increase or a decrease of size [63–76].

**Figure 3:**
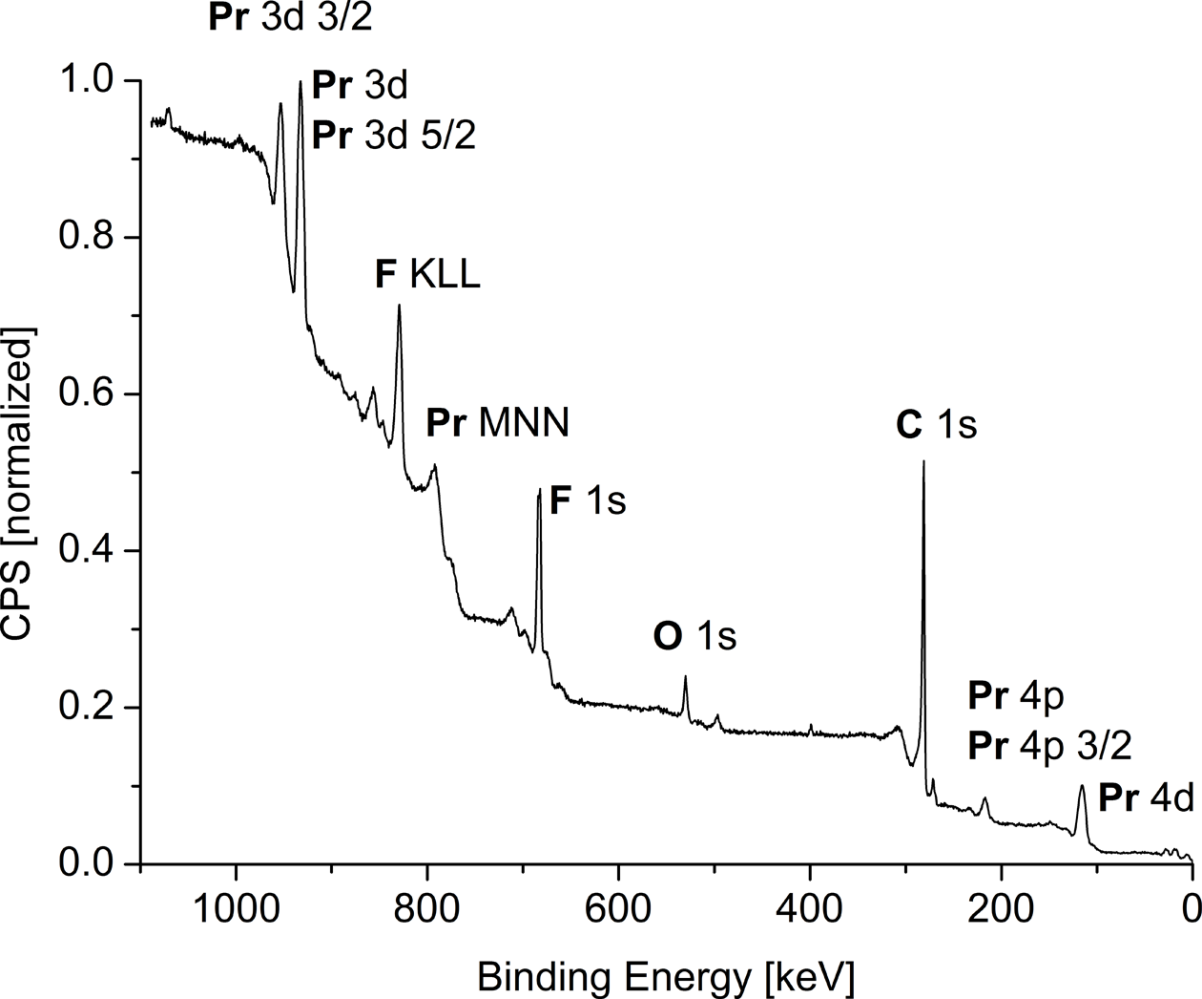
XPS of PrF_3_@TRGO-400 dispersions from [Pr(AMD)_3_] in [BMIm][BF_4_].

XPS measurements (Figure 3, Figures S6–S10 and S12–S15 in Supporting Information File 1) can be used to further support the formation of metal fluorides. The measured electron binding energies of the metals agree with those of the metals in the oxidation states +2 (Fe, Co) or +3 (Pr, Eu) and significantly higher than those of the state M^0^. The F 1s binding energy agrees with those found for metal fluorides, which is 2–5 keV lower than for organic fluorides (Table 2, Tables S9–S11 in Supporting Information File 1).

**Table 2:** Comparison of XPS binding energies.^a^

PrF_3_@TRGO–400 binding energies [keV]			

element	measured	Pr^0^ metal	Pr^3+^ oxidation state [77–78]
Pr 3d 5/2	934.3	932	933–933.5

	measured	metal fluorides	organic fluorides [77–78]
F 1s	686.3	684–685.5	688–689

^a^charge calibration: C 1s 284.8 eV; comparison of XPS binding energies in other MF*_x_*@TRGO samples is given in Tables S9–S11 in Supporting Information File 1.

TRGO still possesses oxygen functionalities on the surface. The presence of oxygen functionalities at the graphene surface provides reactive sites for the nucleation and growth of metal nanoparticles. The nucleation and growth mechanism depends on the degree of oxygen functionalization at the graphene surface sheets, such that no nanoparticles are obtained at totally reduced graphene surfaces [7,79]. TRGO is generally regarded as a good base material for obtaining highly loaded nanoparticle–graphene hybrid materials, because of its surface functionalization [80]. A distinct possibility is the formation of hydrogen bonds between the metal-fluoride nanoparticles and the hydroxyl groups at the TRGO surface.

**Figure 4:**
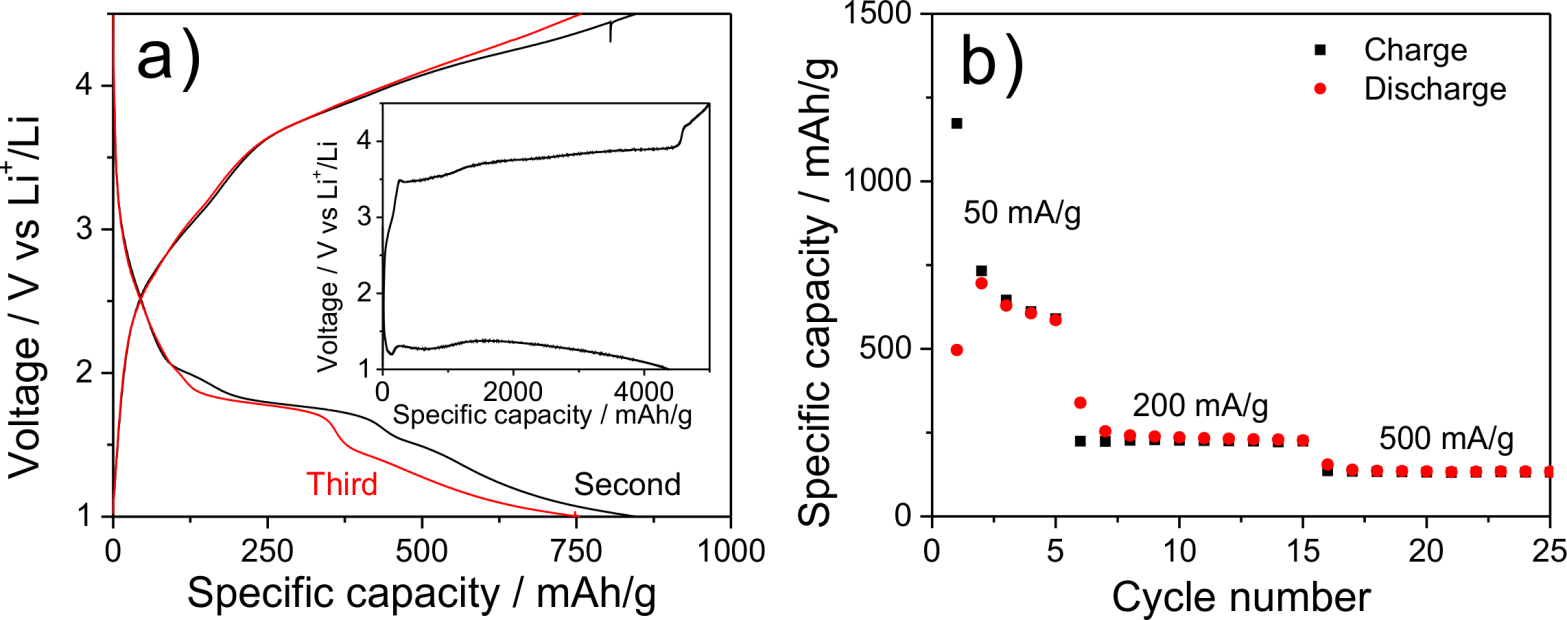
The electrochemical performance of FeF_2_@TRGO-400 as cathode material for lithium-ion batteries. (a) The galvanostatic charge/discharge profiles at a current of 50 mA/g. The inset is the profile of the first cycle. (b) The rate performance after an activation over three cycles.

Batteries based on nanosized materials would yield, for example, short charging time, long lifetime and high capacity [81–82]. Li et al. showed that the use of FeF_2_ NPs, instead of macroscopic LiFeF_3_, led to a significant improvement in the performance of the batteries [83]. The IL [BMIm][BF_4_] was described as the fluoride source for the formation of FeF_3_ NPs and their stabilization medium [84]. Iron fluorides were recognized as promising cathode materials for lithium-ion batteries due to the higher energy density compared to current cathode materials. Iron fluorides can undergo a conversion reaction delivering a theoretical capacity of 712 mAh/g for FeF_3_ and 571 mAh/g for FeF_2_ [85–87]. Here, the electrochemical performance of the obtained FeF_2_@TRGO as cathode materials were evaluated by galvanostatic charge/discharge profiles as shown in Figure 4.

In the first charge/discharge profiles, there is a dip before the plateau, which is normally observed in pure FeF_2_ electrodes [88–89]. This feature corresponds to the conversion reaction of FeF_2_ to Fe^0^ and LiF. The plateau potential is around 1.3 V, far lower than the equilibrium potential of 2.6 V, which can be due to the restricted process kinetics. At the following discharging curves, the plateau potential increases to 1.8 V due to improved process kinetics caused by the reduced particle size during the cycling [88–89]. The region before the plateau is corresponding to the reduction reaction from Fe^3+^ to Fe^2+^, while the part following the plateau is probably caused by the interfacial charge storage at the interface between nanosized Fe and the electrolyte LiF, analogous to the phenomena in RuO_2_ proposed by Maier et al. [90]. The capacity is around 800 mAh/g. During the charging process, there are several oxidation processes, which can be ascribed to the reaction of Fe^0^ to Fe^2+^ (at a potential lower than 3.5 V) and Fe^2+^ to Fe^3+^ (at a potential higher than 3.5 V) [88]. The large voltage hysteresis between discharging and charging process can be attributed to sluggish process kinetics, including, for example, phase evolution and the spatial distribution of immediate phases [88–91]. At the first discharge and charge process, the very high capacity may be caused by the formation of a solid–electrolyte interface. After several cycles at 50 mA/g, the capacity stabilizes to around 500 mAh/g and decreases to 220 and 130 mAh/g with the current density increasing to 200 and 500 mA/g, respectively. The results indicate the good rate performance of FeF_2_@TRGO-400.

## Conclusion

We were able to confirm the successful decomposition of transition-metal amidinates [M{MeC[N(iPr)]_2_}*_n_*] [M(AMD)*_n_*; M = Fe(II), Co(II), Pr(III)] as well as tris(2,2,6,6-tetramethyl-3,5-heptanedionato)europium, Eu(dpm)_3_, to metal-fluoride nanoparticles in the ionic liquid [BMIm][BF_4_]. We describe a simple method for the support of largely isolated metal-fluoride nanoparticles on different types of TRGO, differing in the reduction temperatures (300, 400 or 750 °C) from graphite oxide and in the presence of sulfur functionalities. The nanoparticles exhibited mostly diameters of less than 30 nm. For cobalt it was only possible to support non-aggregated CoF_2_ particles on TRGO-SH. The results support the advantages of the metal-organic precursor concept based on metal amidinates together with non-conventional solvents and microwave-assisted pyrolysis [92–94]. Galvanostatic charge/discharge profiles of FeF_2_@TRGO-400 indicate a good rate performance of the composite material, e.g., capacities of 220 and 130 mAh/g at current densities of 200 and 500 mA/g, respectively.

## Experimental

All syntheses were carried out under nitrogen or argon using Schlenk techniques, since the amidinates are hygroscopic and air sensitive. 1,3-Diisopropylcarbodiimide (>99%), iron(II) chloride (>98%), cobalt(II) chloride (>99%), methyllithium, 1-chlorobutane (>99%) and 1-methylimidazole(>99%), were purchased from Sigma-Aldrich and used without further purification. Tris(2,2,6,6-tetramethyl-3,5-heptanedionato)europium(III) (>99 %) was obtained from Alfa Aesar and was dried under high vacuum (10^−3^ mbar) for several days. Lithium amidinate was synthesized by deprotonation and methylation of 1,3-diisopropylcarbodiimide with methyllithium and subsequently reaction with metal halides according to literature procedures [95–96].

The ionic liquid [BMIm][BF_4_] was synthesized by reacting 1-methylimidazole with 1-chlorobutane to yield [BMIm][Cl]. [BMIm][Cl] reacted with HBF_4_ to give [BMIm][BF_4_] [64]. Following the washing procedure with water the IL was dried under ultra-high vacuum (10^−7^ mbar) at 60 °C for several days.

Thermally reduced graphene oxide (TRGO) was prepared in a two-step oxidation/thermal reduction process using natural graphite (type KFL 99.5 from AMG Mining AG, former Kropfmühl AG, Passau, Germany) as raw material. The graphite oxidation procedure of Hummers and Offeman [6] was employed. All TRGOs, differing in the reduction temperatures (300, 400 or 750 °C) from graphite oxide and in the presence of sulfur functionalities, were obtained from the group of Prof. Rolf Mülhaupt, University of Freiburg. For the TRGO analyses see Supporting Information File 1 (Figures S1–S3, Tables S1–S7).

X-ray photoelectron spectroscopy, XPS-(ESCA), measurements were performed with a Fisons/VG Scientific ESCALAB 200X spectrometer, operating at room temperature at a pressure of 1.0 × 10^−8^ bar and a sample angle of 30°. Spectra were recorded using polychromatic Al Kα excitation (14 kV, 20 mA) at an emission angle of 0°. Calibration was carried out by recording spectra with Al Kα X-rays from clean samples of copper, silver and gold at 20 eV and 10 eV pass energies and comparison with reference values.

Powder X-ray diffractograms, PXRDs, were measured at ambient temperature on a Bruker D2 Phaser using a flat sample holder and Cu Kα radiation (λ = 1.54182 Å, 35 kV). The samples had been precipitated with acetonitrile from the NP/IL dispersion and washed several times with acetonitrile. PXRDs were measured for 1 h. Small shifts in PXRD patterns are not uncommon for nanoparticles. A number of effects can be considered for such shifts including a range of stoichiometric composition, partly inhomogeneous element distribution, defects such as stacking and twin faults and nanosized crystalline domains being much smaller than the bulk reference material causing lattice contraction or expansion and strain [97–101].

The HR-TEM imaging was performed on a FEI Tecnai G2 F20 electron microscopy operated at 200kV accelerating voltage [102]. Digital images were recorded by a Gatan UltraScan 1000P detector. Samples were prepared using 200 μm carbon-coated copper grids or gold grids. The size distribution was determined manually or with the aid of the Gatan DigitalMicrograph software from at least 50 individual particles.

HR-TEM EDX spectroscopy was also performed on a FEI Tecnai G2 F20 with a high-angle energy-dispersive X-ray detector providing a resolution of 136 eV or better for Mn Kα radiation. The exposure time of individual EDX spectra was 3 min.

Metal-fluoride nanoparticles were prepared in a nitrogen atmosphere. 10 mg of the TRGO and the weighted amount of metal-amidinate powder or [Eu(dpm)_3_] were suspended at room temperature in the dried ionic liquid in a microwave vial in a glove box. The vial was closed with a cramp cap in the glove box before being taken out. The mass of the metal precursor was set for a 0.5 wt % M-NP dispersion in IL. The vial with the reaction mixture was placed in a microwave (CEM, Discover) and irradiated for 10 min (Co) or 15 min (Fe, Pr, Eu) at a power of 50 W to a temperature of 220 °C.

Examples of selected area electron diffraction (SAED) patterns (Figures S4 and S6 in Supporting Information File 1) have been recorded with an FEI Titan 80-300 TEM [103], operated at 300 kV accelerating voltage. The area selection was achieved with a round aperture placed in the first intermediate image plane with a corresponding diameter of 0.64 µm in the object plane. For each acquisition a sample region with a significant amount of material was placed inside the aperture. The objected was illuminated with wide-spread parallel beam obtaining focused diffraction patterns. The diffraction images were calibrated with Debye–Scherrer patterns recorded from a gold reference sample (S106, Plano GmbH, Wetzlar, Germany).

For the electrochemical measurements, the working electrodes were prepared by coating a slurry composed of 75 wt % FeF_2_-TRGO, 15 wt % active carbon and 10 wt % PVDF in NMP on an aluminum foil. A half-cell was assembled in Ar-filled glovebox with lithium foil as counter electrode and 1 M LiFeF_6_ in ethylene carbonate/ethylmethyl carbonate (50:50) as electrolyte. The galvanostatic charge/discharge profiles were collected on a Maccor battery cycler with cut-off potentials of 4.5 and 1.0 V vs Li^+^/Li.

## Supporting Information

Information about the synthesis of TRGO and TRGO-SH, the analysis of TRGO-300, -400, -750 and -SH, and an overview of all samples.

File 1 Additional experimental data.
