# Supplementary material for: Synthesis of metal-fluoride nanoparticles supported on thermally reduced graphite oxide
Source: Beilstein J Nanotechnol. 2017 Nov 22;8:2474–83. doi: 10.3762/bjnano.8.247 (PMC5704767; doi:10.3762/bjnano.8.247)
Supplement: File 1 — Additional experimental data. [file Beilstein_J_Nanotechnol-08-2474-s001.pdf]

Supporting Information

for

Synthesis of metal-fluoride nanoparticles supported on thermally reduced graphite oxide

Alexa Schmitz[1], Kai Schütte[1], Vesko Ilievski[1], Juri Barthel[2], Laura Burk[3], Rolf Mülhaupt[3], Junpei Yue[4], Bernd Smarsly[4] and Christoph Janiak*,§,1

Address: [1]Institut für Anorganische Chemie und Strukturchemie, Heinrich-Heine-Universität Düsseldorf, 40204 Düsseldorf, Germany; [2]Gemeinschaftslabor für Elektronenmikroskopie RWTH-Aachen, Ernst Ruska-Centrum für Mikroskopie und Spektroskopie mit Elektronen, D-52425 Jülich, Germany; [3]Freiburg Materials Research Center and Institute for Macromolecular Chemistry, Albert-Ludwigs-University Freiburg, 79104 Freiburg, Germany and [4]Physikalisch-Chemisches Institut, Justus-Liebig-Universität Gießen, 35392 Gießen, Germany

Email: Christoph Janiak* - janiak@uni-duesseldorf.de

* Corresponding author

§Fax: +49-211-81-12287; Tel: +49-211-81-12286

Additional experimental data

Synthesis of TRGO and TRGO-SH

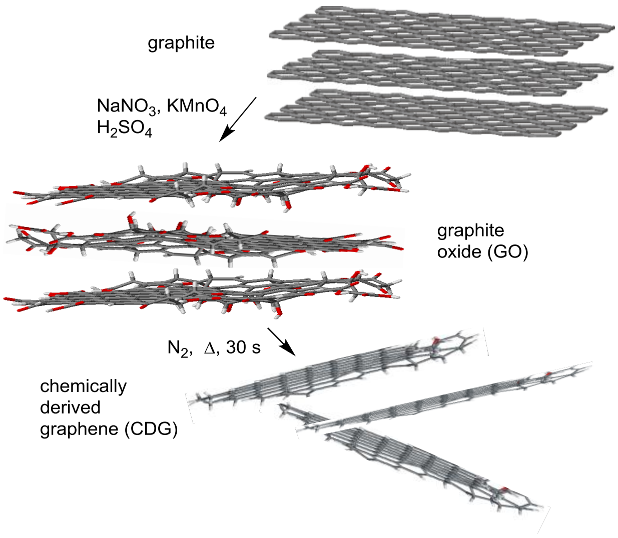

Scheme S1: Synthesis of chemically derived graphene (CDG)/ thermally reduced graphite oxide (TRGO) from natural graphite over graphite oxide (adapted from [1]).

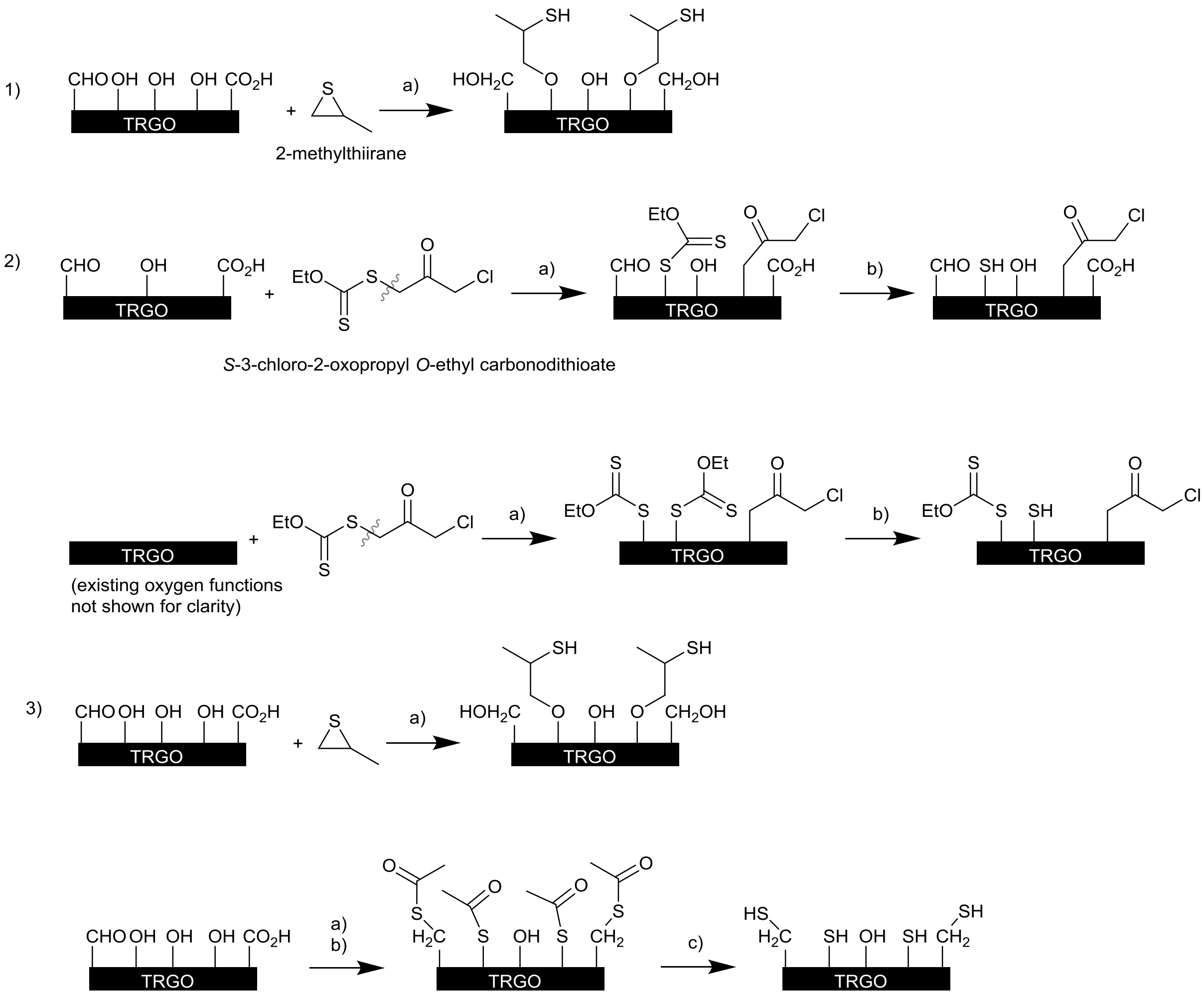

Scheme S2: Synthesis of TRGO-SH. Three different routes for the synthesis of TRGO-SH from TRGO-400 (adapted from [2]).

Analysis of TRGO-300, -400, -750 and –SH

TRGO-300

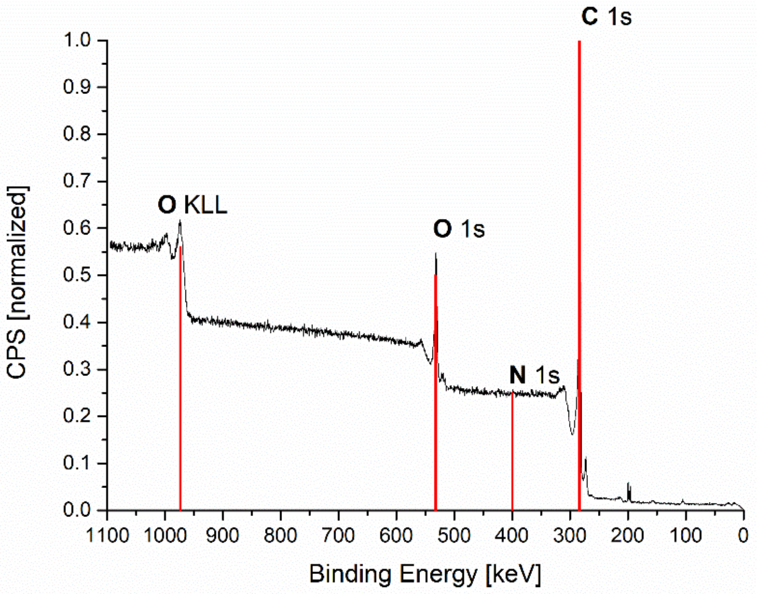

Figure S1: Photoelectron spectrum of TRGO-300. The red bars are a guide to the eye on the binding energy axis.

Table S1: XPS quantification of TRGO-300.

name	position	area	atom %	R.S.F.
O 1s	532	111030	14	2.93
C 1s	283	223791	86	1

In the XP spectra a clear oxygen and carbon signal was seen. Quantification of oxygen against carbon showed that TRGO-300 contains 14 atom % oxygen.

Table S2: CHN analysis of TRGO-300.

Sample	C [wt %]	H [wt %]	S [wt %]
TRGO-300	79.86	0.77	0.55

The sulfur impurity is believed to be derived from graphite.

The BET surface of TRGO-300 was 430 m^2/g.

TRGO-400

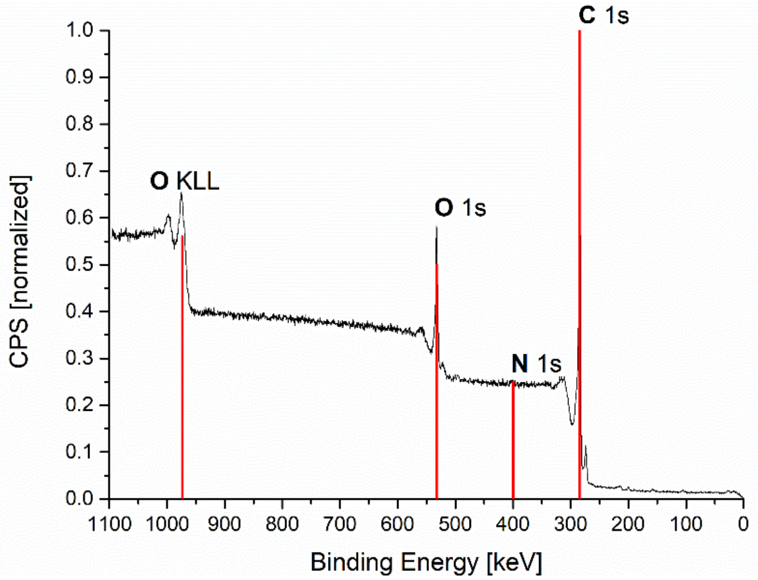

Figure S2: Photoelectron spectrum of TRGO-400. The red bars are a guide to the eye on the binding energy axis.

Table S3: XPS quantification of TRGO-400.

name	position	area	atom %	R.S.F.
O 1s	533	127192	15	2.93
C 1s	284	248465	85	1

In the XP spectra a clear oxygen and carbon signal was seen. Quantification of oxygen against carbon showed that TRGO-300 contains 15 atom % oxygen.

Table S4: CHN analysis of TRGO-400.

sample	C [wt %]	H [wt %]	S [wt %]
TRGO-400	80.93	0.8	0.73

The S impurity is believed to be derived from graphite.

The BET surface of TRGO-400 was 450 m^2/g.

TRGO-750

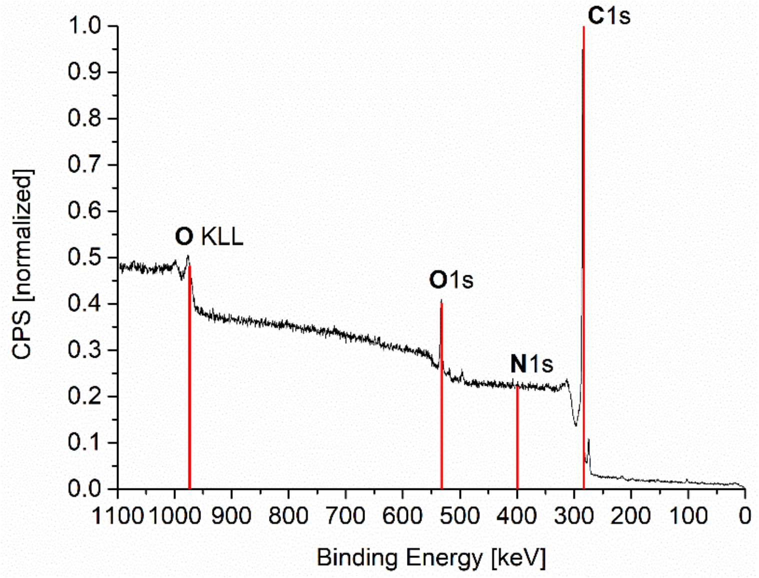

Figure S3: Photoelectron spectrum of TRGO-400. The red bars are a guide to the eye on the binding energy axis.

Table S5: XPS quantification of TRGO-750.

name	position	area	atom %	R.S.F.
O 1s	533	41954	9	2.93
C 1s	284	150049	91	1

In the XP spectra a clear oxygen and carbon signal was seen. Quantification of oxygen against carbon showed that TRGO-300 contains 9 atom % oxygen.

Table S6: CHN analysis of TRGO-750.

sample	C [wt %]	H [wt %]
TRGO-750	82.39	0.81

The BET surface of TRGO-750 was 520 m^2/g.

TRGO-SH

Table S7: CHN analysis of TRGO-SH.

sample	C [wt %]	H [wt %]	S [wt %]
TRGO-SH	54.77	1.48	0.96

The BET surface of TRGO-SH was 189 m^2/g.

Overview of all samples

MFx@TRGO-300:

<u>FeF_2@TRGO-300</u>

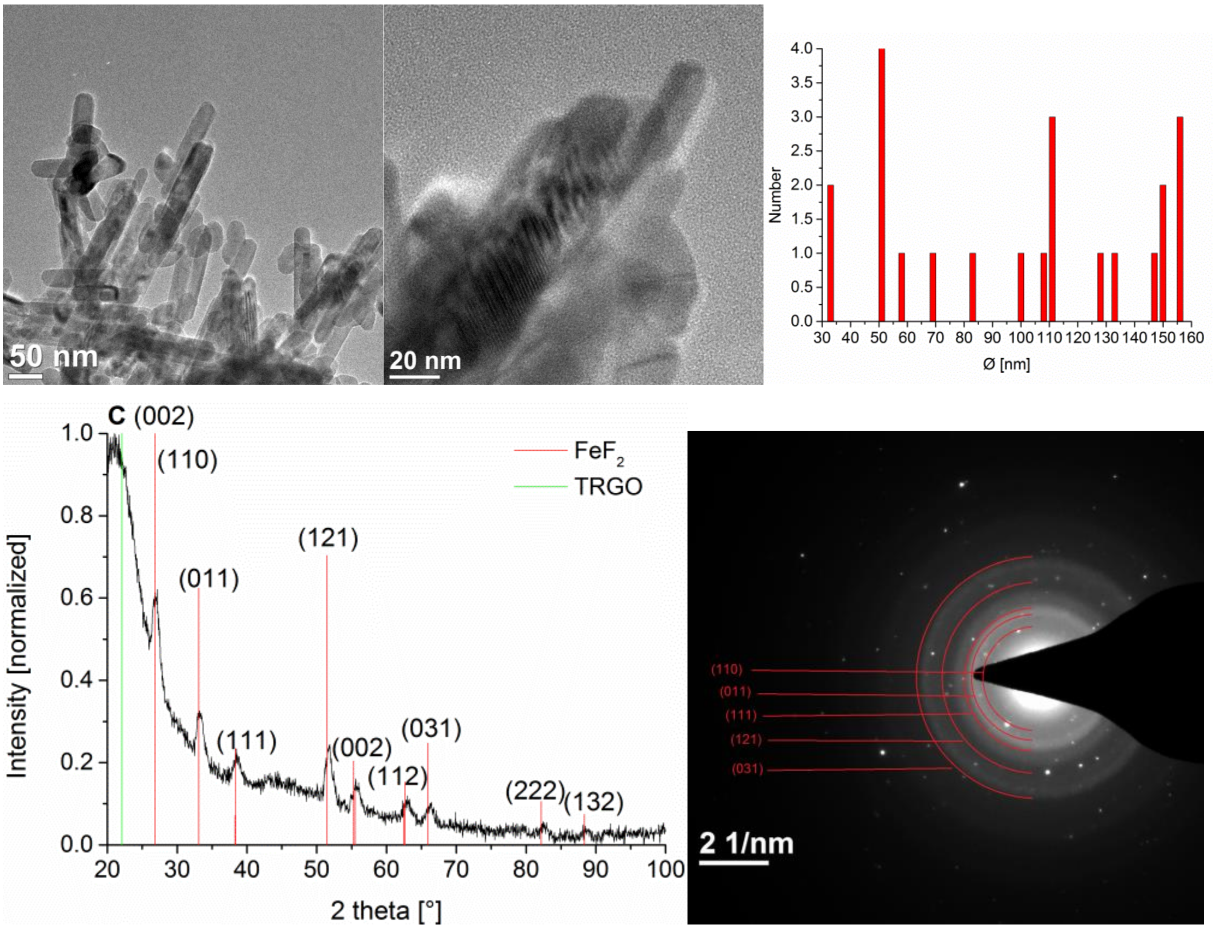

Figure S4: TEM and size distribution (top), PXRD and SAED (bottom, FeF_2–reference reflections in red from COD 9009074) of 0.5 wt % FeF_2-NPs in [BMIm][BF_4] @TRGO–300 from $Fe(AMD)_2$.

CoF$_2$@TRGO-300

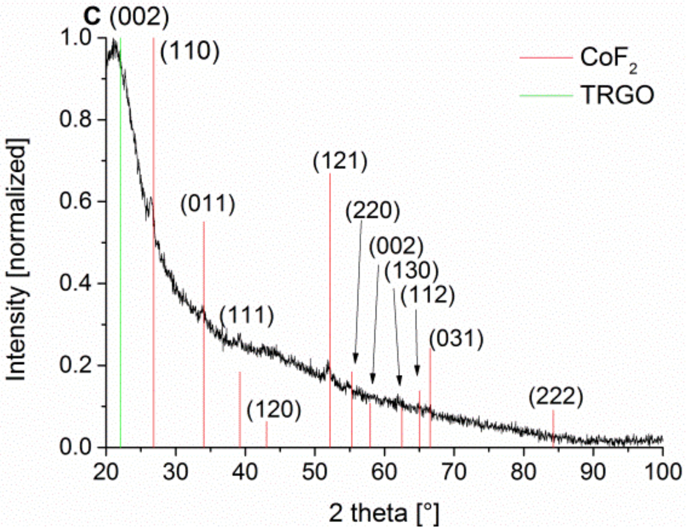

Figure S5: PXRD (CoF_2 – reference reflections in red from COD 9009073) of 0.5 wt % CoF_2–NPs in [BMIm][BF_4] @TRGO–300 from Co(AMD)$_2$.

PrF$_3$@TRGO-300

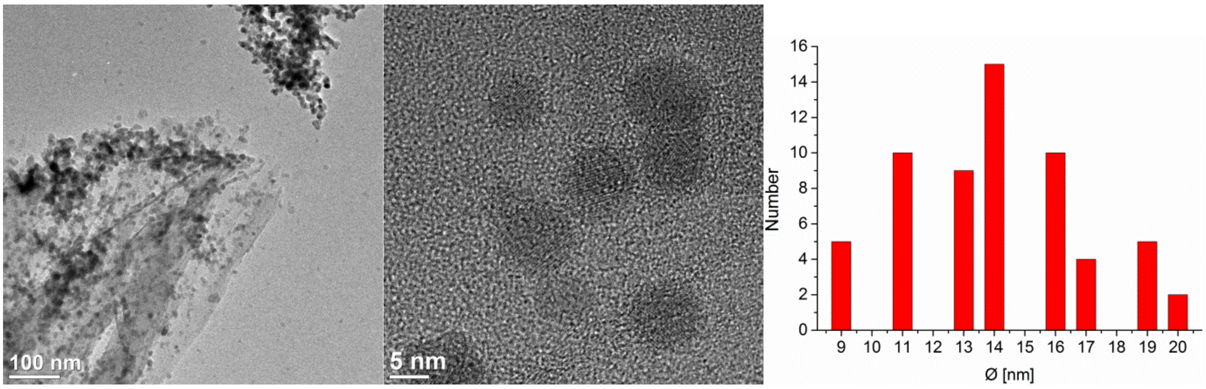

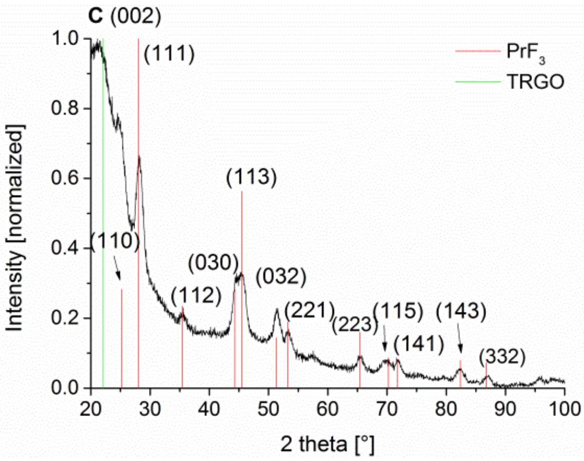

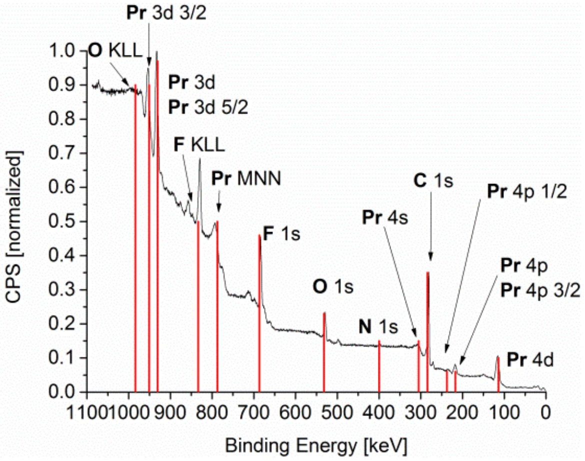

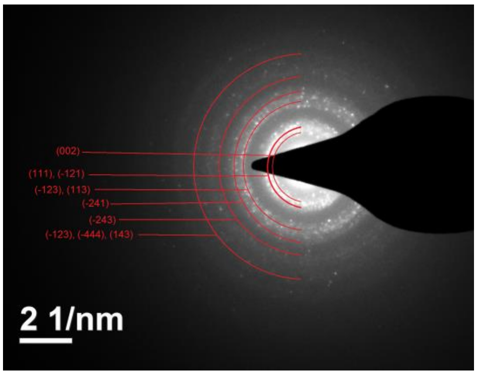

Figure S6: TEM and size distribution (top), PXRD and XPS (middle, PrF_3- reference reflections in red from COD 1010984) and SAED (bottom) of 0.5 wt % PrF_3-NPs in [BMIm][BF_4] @TRGO–300 from $Pr(AMD)_3$. XPS: The red bars are a guide to the eye on the binding energy axis.

<u>EuF_3@TRGO-300</u>

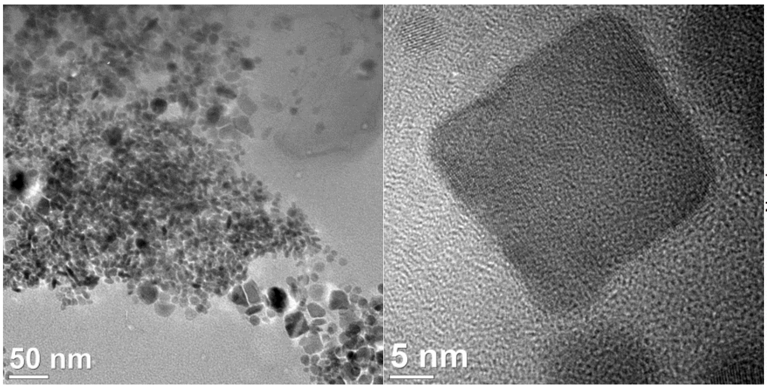

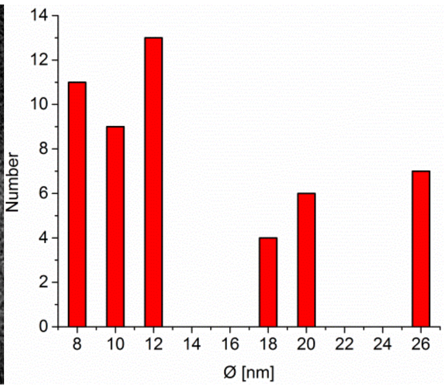

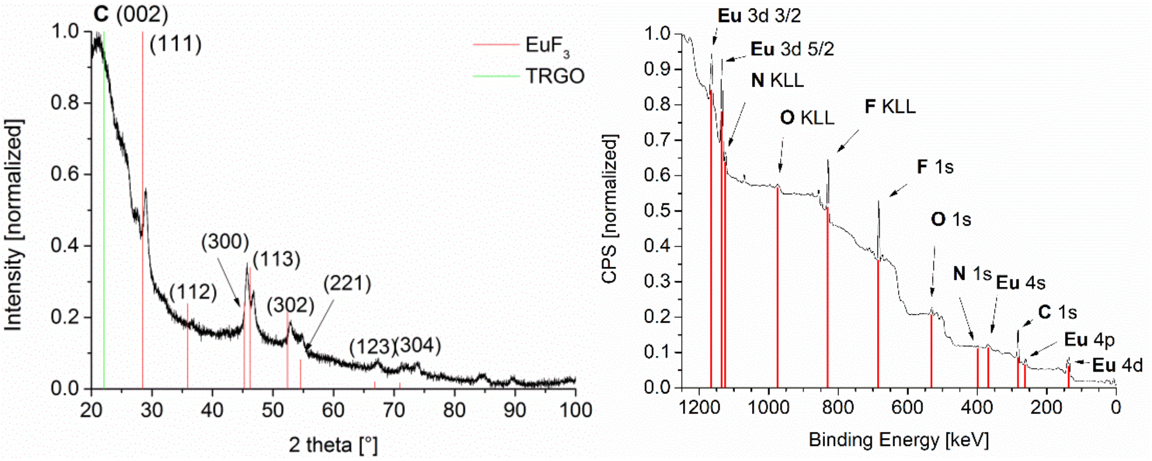

Figure S7: TEM and size distribution (top), PXRD and XPS (bottom, EuF_3- reference reflections in red from ICDD 33–0373) of 0.5 wt % EuF_3-NPs in [BMIm][BF_4] @TRGO–300 from Eu(dpm)$_3$. XPS: The red bars are a guide to the eye on the binding energy axis.

Table S8: d-spacing references for FeF_2 and PrF_3 from the literature compared with the measured d-spacing.

d-spacing reference (hkl) [FeF_2 COD: 9009074]	*d*-spacing measured	*d*-spacing reference (hkl) [PrF_3 COD: 1010984]	*d*-spacing measured
3.32 (110)	3.30	3.61 (002)	3.63
2.70 (011)	2.71	3.17 (111/–121)	3.14
2.34 (111)	2.38	1.99 (–123/113)	1.99
1.77 (121)	1.78	1.71 (–241)	1.70
1.41 (031)	1.42	1.42 (–243)	1.44
		1.16 (–153/ –444/ 143)	1.14

Table S9: Comparison of XPS binding energies.

MF$_x$@TRGO–300 binding energies [keV]<br><br>metal signals			
element	measured	M(0) metal	M(3+) oxidation state [3,4]
Pr 3d 5/2	934.6	932	933–933.5
Eu 3d 5/2	1135.8	1126	3: 1135
F 1s signal			
	measured	metal fluorides	organic fluorides [3,4]
F (in PrF_3)	686	684–685.5	688–689
F (in EuF_3)	684.8		

TRGO-400:

FeF$_2$@TRGO-400

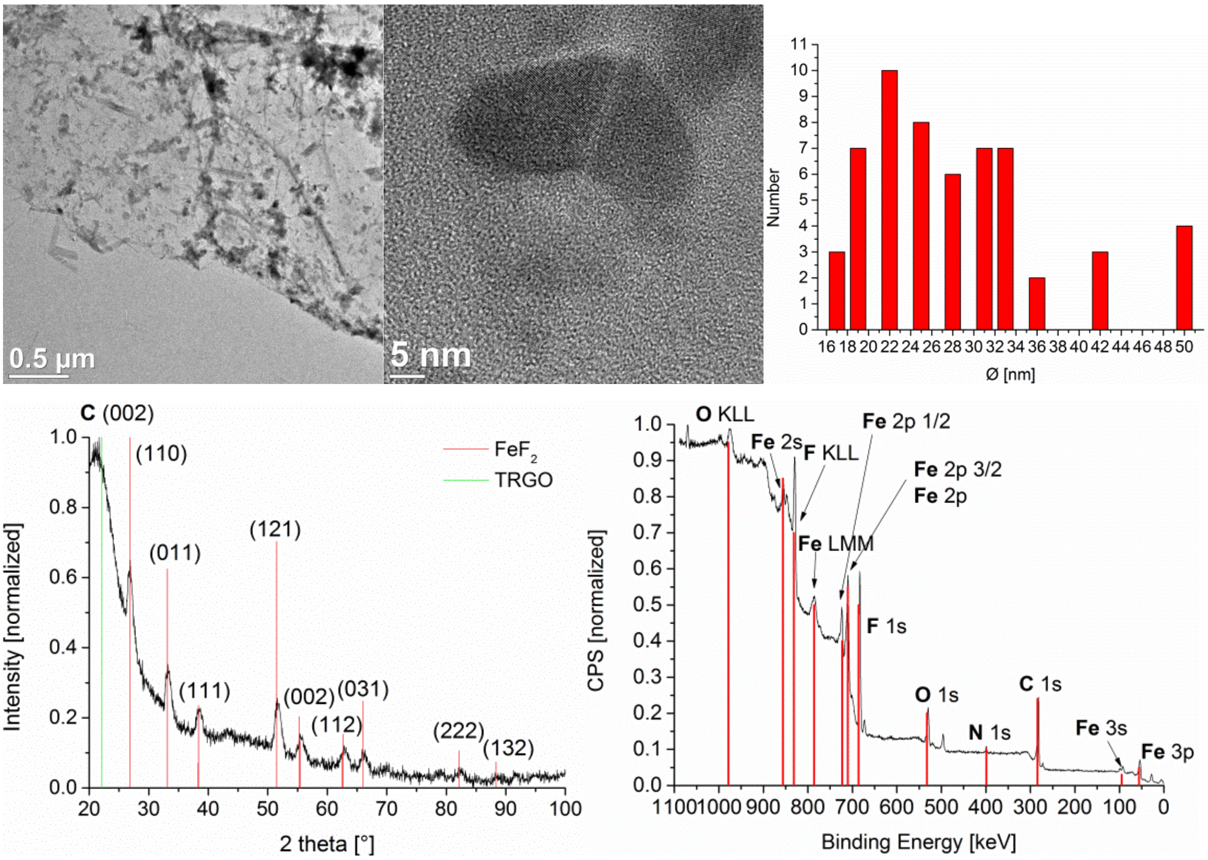

Figure S8: TEM and size distribution (top), PXRD and XPS (bottom, FeF$_2$-reference reflections in red from COD 9009074) of 0.5 wt % FeF$_2$-NPs in [BMIm][BF$_4$] @TRGO–400 from Fe(AMD)$_2$. XPS: The red bars are a guide to the eye on the binding energy axis.

CoF$_2$@TRGO-400

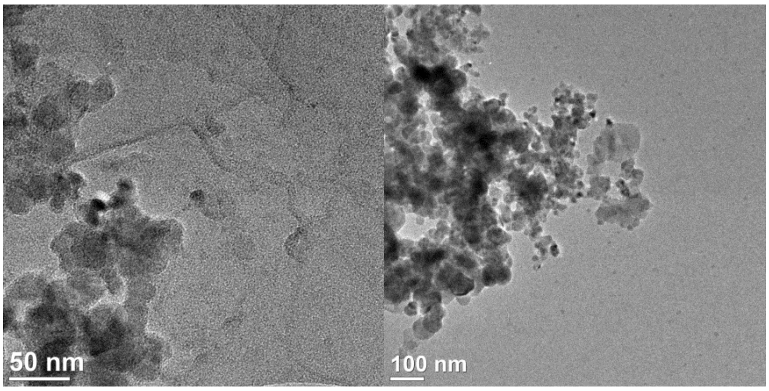

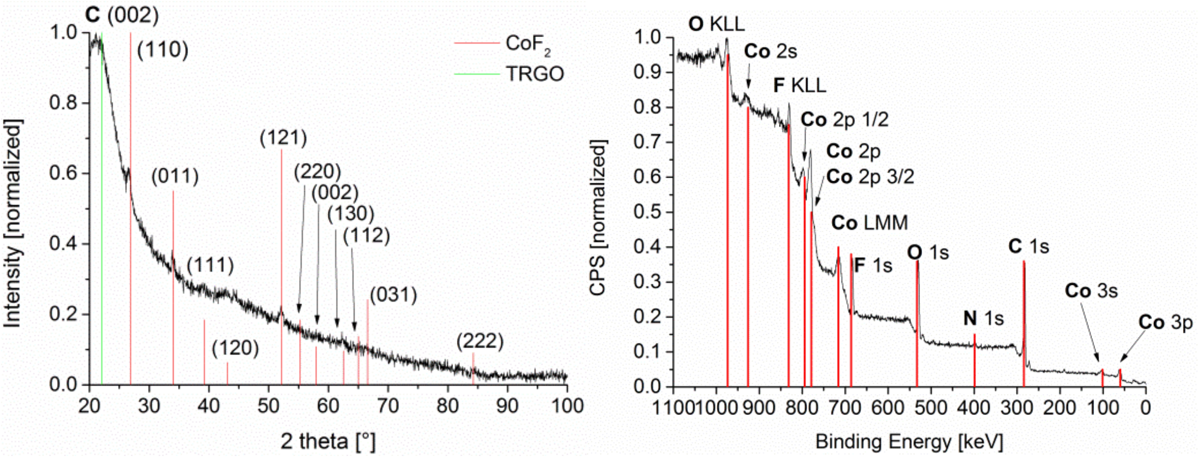

Figure S9: TEM and PXRD (top) CoF_2– reference reflections in red from COD 9009073), XPS (bottom) of 0.5 wt % CoF_2–NPs in [BMIm][BF_4] @TRGO–400 from $Co(AMD)_2$. XPS: The red bars are a guide to the eye on the binding energy axis.

<u>PrF_3@TRGO-400</u>

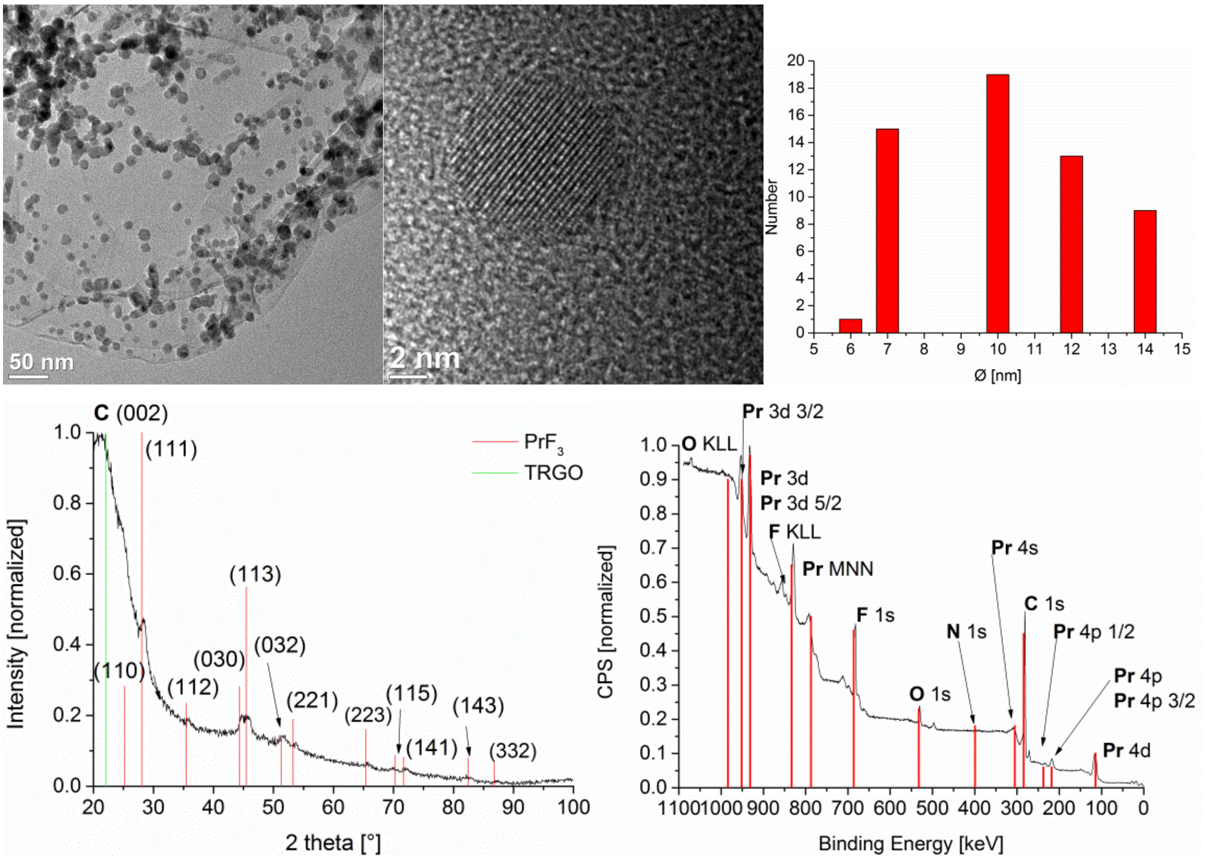

Figure S10: TEM and size distribution (top), PXRD and XPS (bottom, PrF_3-reference reflections in red from COD 1010984) of 0.5 wt % PrF_3-NPs in [BMIm][BF_4] @TRGO–400 from $Pr(AMD)_3$. XPS: The red bars are a guide to the eye on the binding energy axis.

EuF_3@TRGO-400

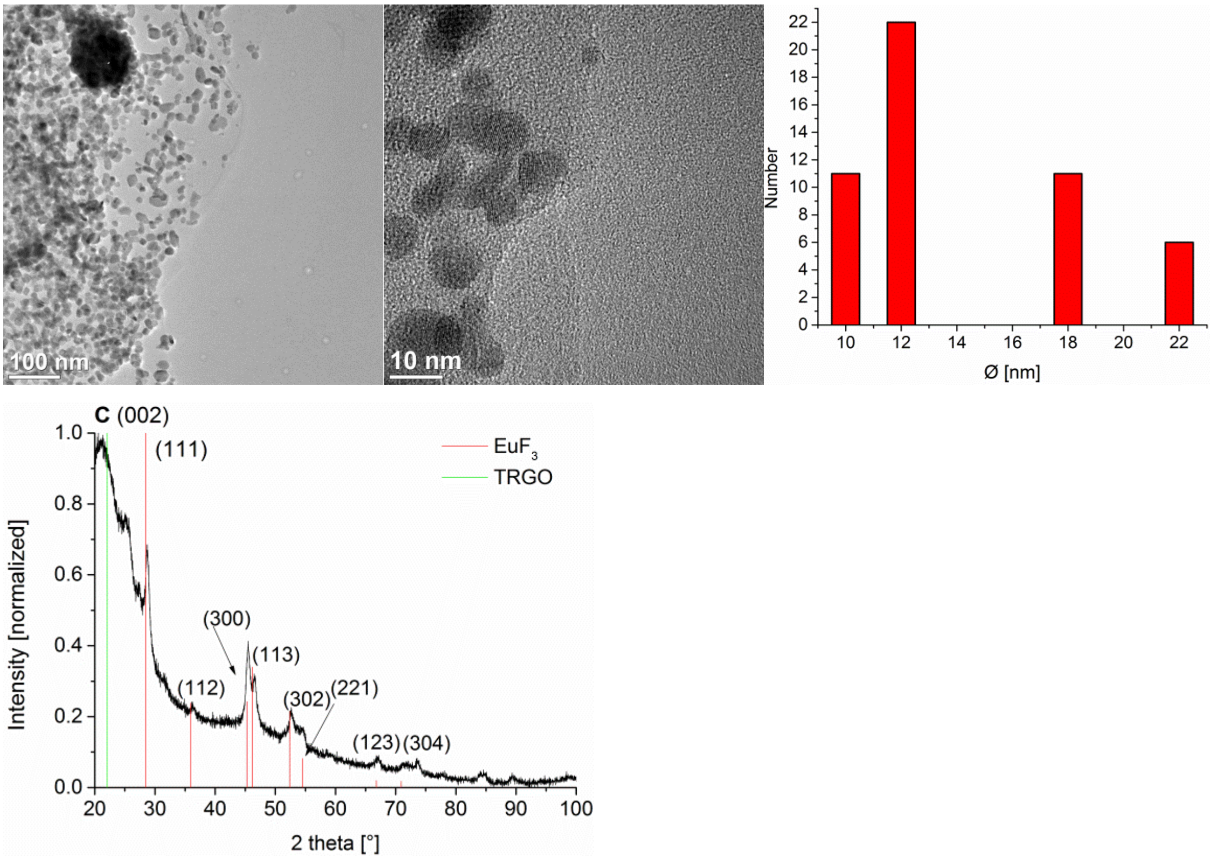

Figure S11: TEM and size distribution (top), PXRD (bottom, EuF_3-reference reflections in red from ICDD 33–0373), of 0.5 wt % EuF_3-NPs in [BMIm][BF_4] @TRGO–400 from $Eu(dpm)_3$.

Table S10: Comparison of the XPS binding energies.

MF$_x$@TRGO–400 binding energies [keV]<br><br>metal signals			
element	measured	M(0) metal	M(+2/+3) oxidation state [3,4]
Fe 2p 3/2	712.5	706.7	+2: 710.4
Co 2p 3/2	783	778.2	+2: 779.7
Pr 3d 5/2	934.3	932	+3: 933–933.5
F 1s signal			
	measured	metal fluorides	organic fluoride [3,4]
F (in FeF_2)	685.6	684–685.5	688–689
F (in CoF_2)	685.9		
F (in PrF_3)	686.3		

TRGO-750:

FeF$_2$@TRGO-750

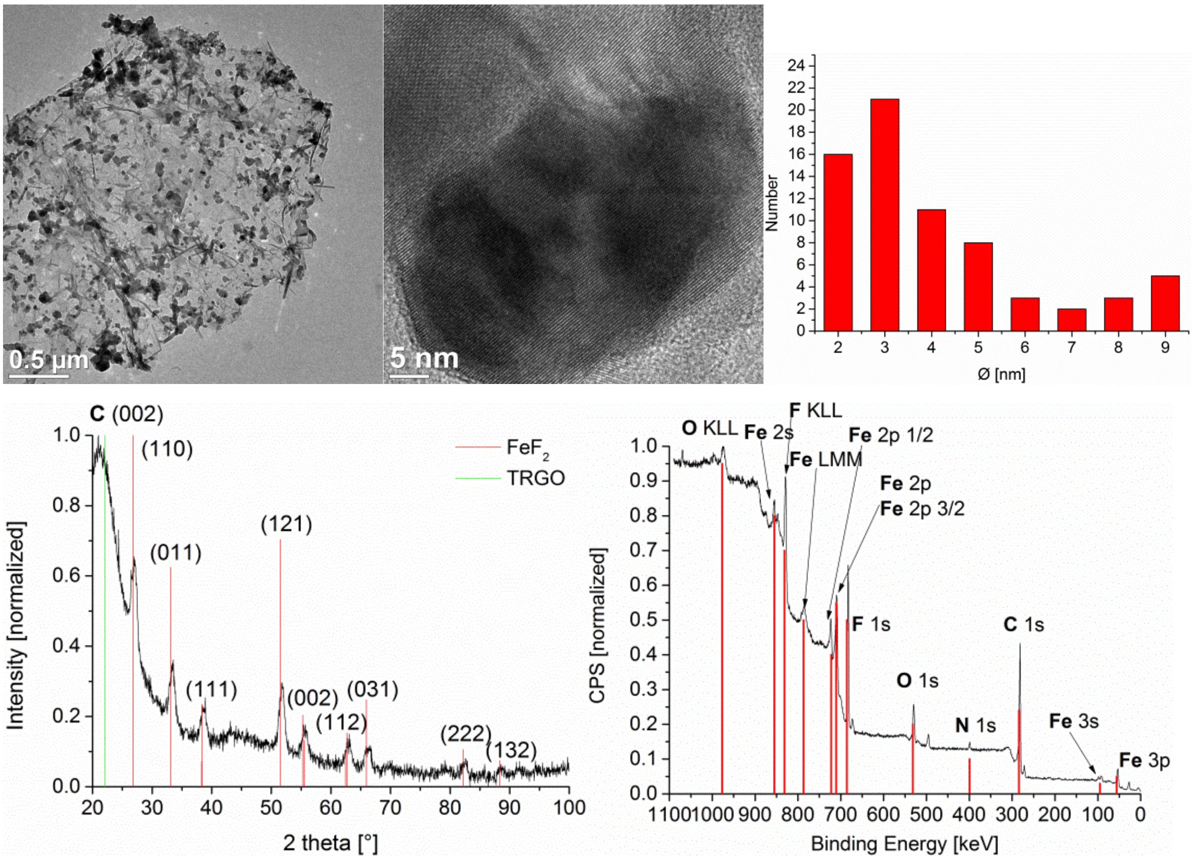

Figure S12: TEM and size distribution (top), PXRD and XPS (bottom, FeF$_2$-reference reflections in red from COD 9009074) of 0.5 wt % FeF$_2$-NPs in [BMIm][BF$_4$] @TRGO–750 from Fe(AMD)$_2$. XPS: The red bars are a guide to the eye on the binding energy axis.

CoF$_2$@TRGO-750

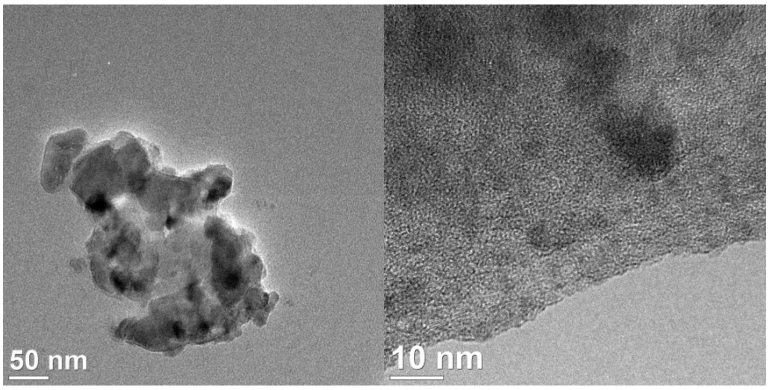

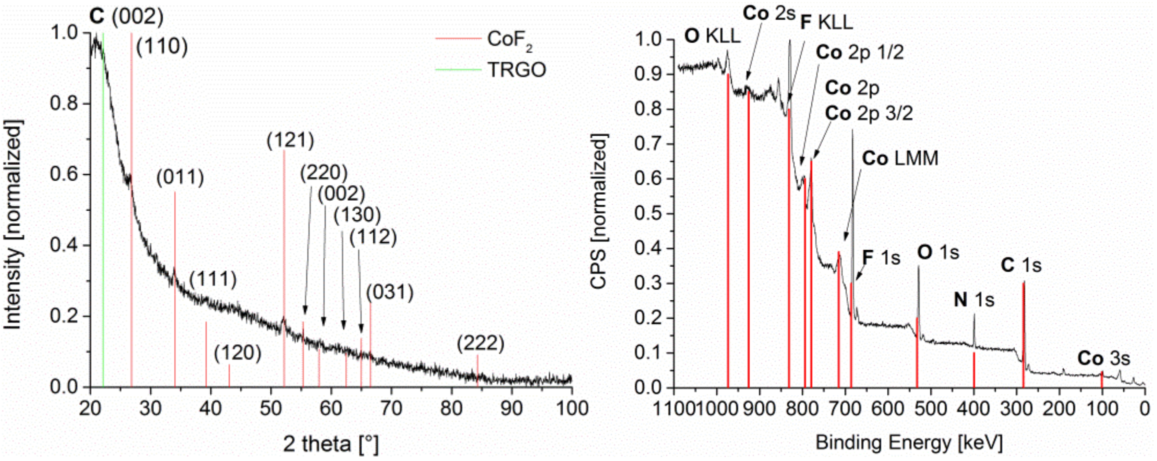

Figure S13: TEM and PXRD (top) CoF_2–reference reflections in red from COD 9009073), XPS (bottom) of 0.5 wt % CoF_2–NPs in [BMIm][BF_4] @TRGO–750 from $Co(AMD)_2$. XPS: The red bars are a guide to the eye on the binding energy axis.

<u>PrF_3@TRGO-750</u>

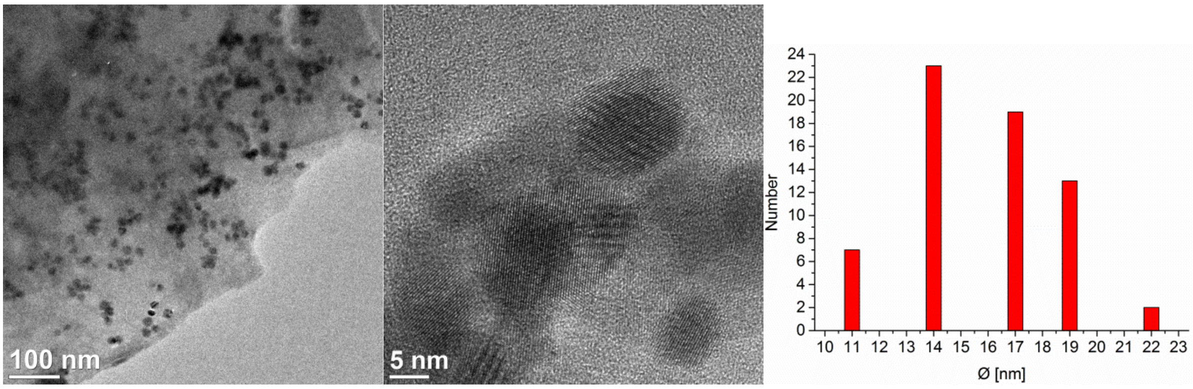

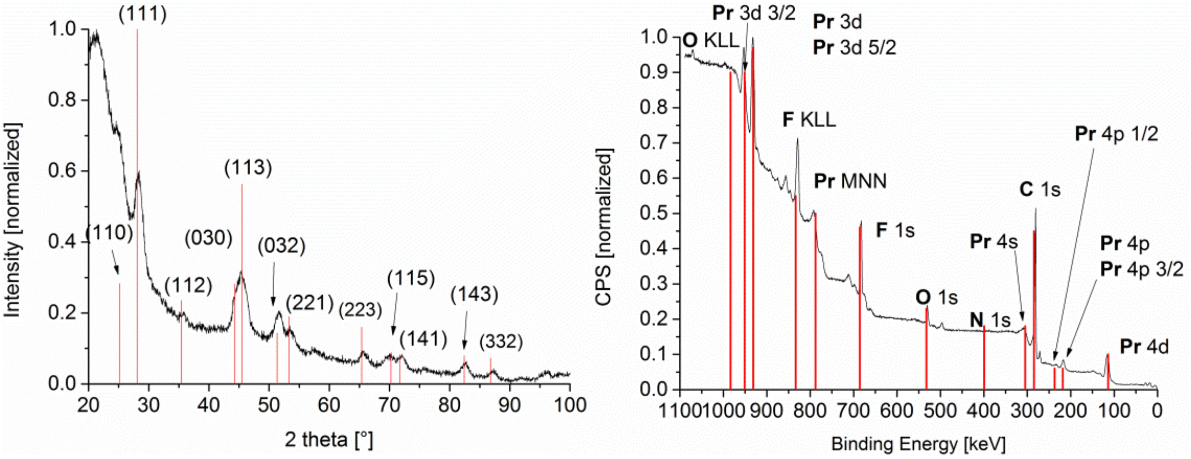

Figure S14: TEM and size distribution (top), PXRD and EDX (middle, PrF_3-reference reflections in red from COD 1010984), XPS (bottom) of 0.5 wt % PrF_3-NPs in [BMIm][BF_4] @TRGO–750 from $Pr(AMD)_3$. XPS: The red bars are a guide to the eye on the binding energy axis.

<u>EuF_3@TRGO-750</u>

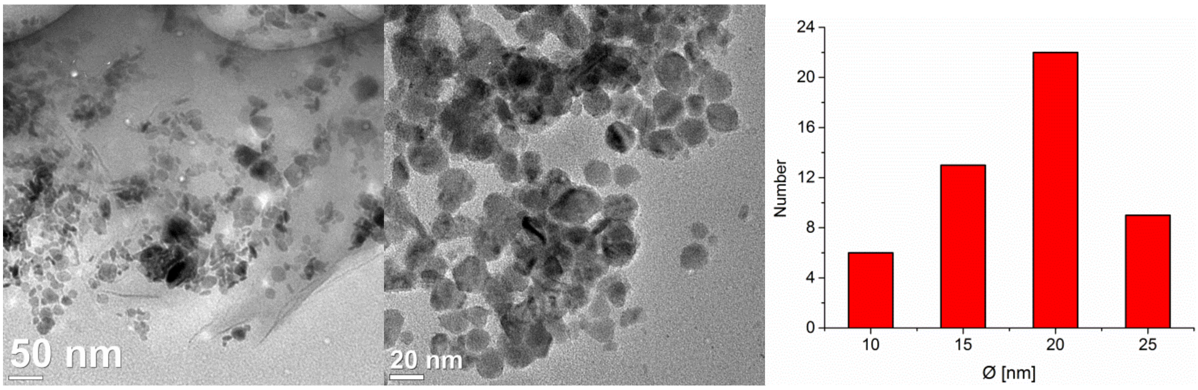

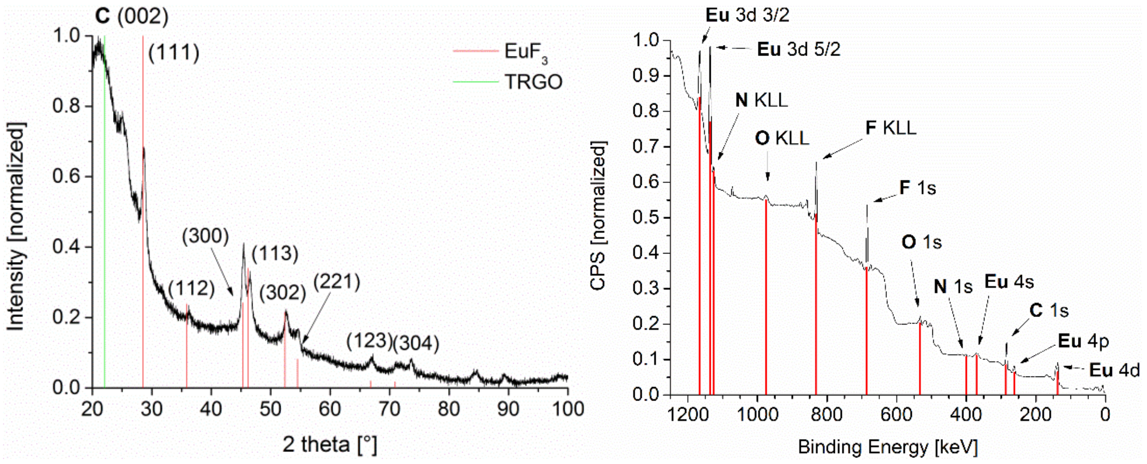

Figure S15: TEM and size distribution (top), PXRD and XPS (bottom, EuF_3-reference reflections in red from ICDD 33–0373) of 0.5 wt % EuF_3-NPs in [BMIm][BF_4] @TRGO–750 from $Eu(dpm)_3$. XPS: The red bars are a guide to the eye on the binding energy axis.

Table S11: Comparison of the XPS binding energies.

MF_x@TRGO–750 binding energies [keV]<br>metal signals			
element	measured	M(0) metal	M(+2/+3) oxidation state [3,4]
Fe 2p 3/2	712.6	706.7	+2: 710.4
Co 2p 3/2	781.7	778.2	+2: 779.7
Pr 3d 5/2	935.5	932	+3: 933–933.5
Eu 3d 5/2	1136.2	1126	+3: 1135
F 1s signal			
	measured	metal fluorides	organic fluorides [3,4]
F (in FeF_2)	685.5	684–685.5	688–689
F (in CoF_2)	685.2		
F (in PrF_3)	686		
F (in EuF_3)	685.5		

TRGO-SH:

FeF$_2$@TRGO-SH

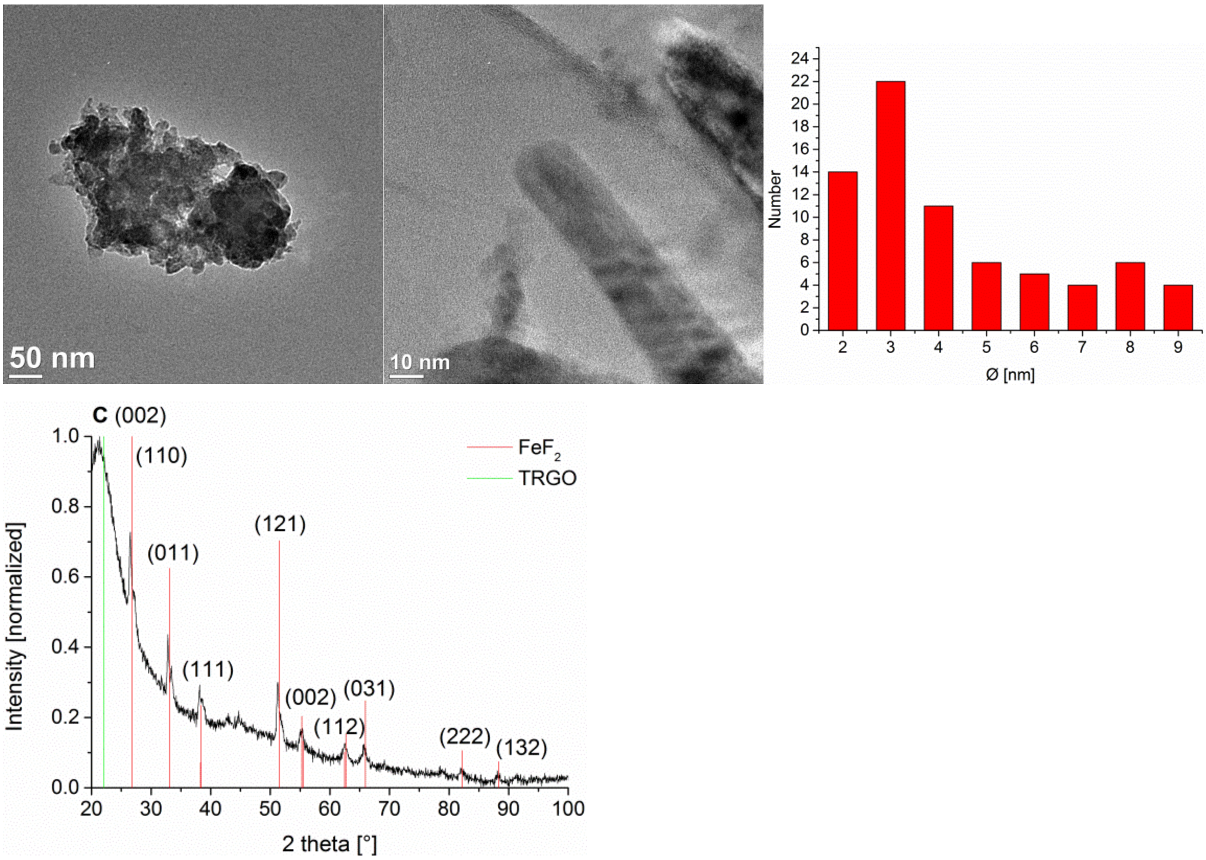

Figure S16: TEM and size distribution (top), PXRD (bottom, FeF$_2$-reference reflections in red from COD 9009074) of 0.5 wt % FeF$_2$-NPs in [BMIm][BF$_4$] @TRGO–SH from Fe(AMD)$_2$.

CoF$_2$@TRGO-SH

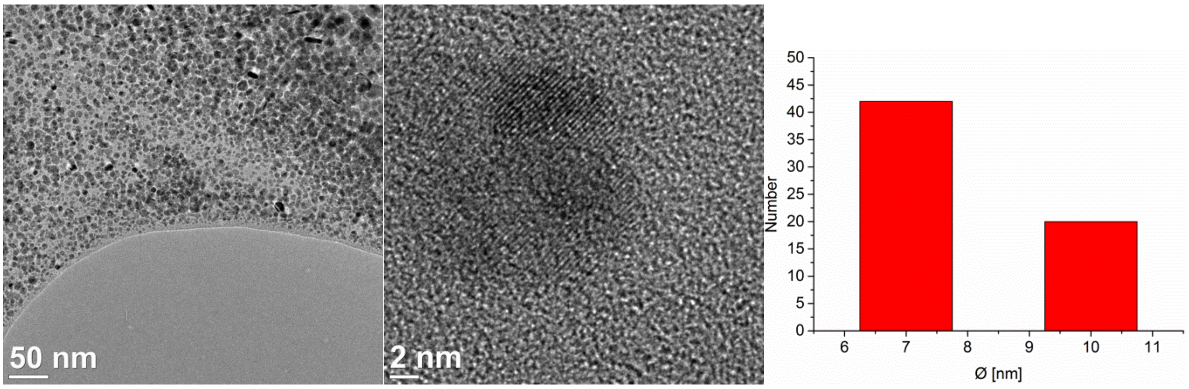

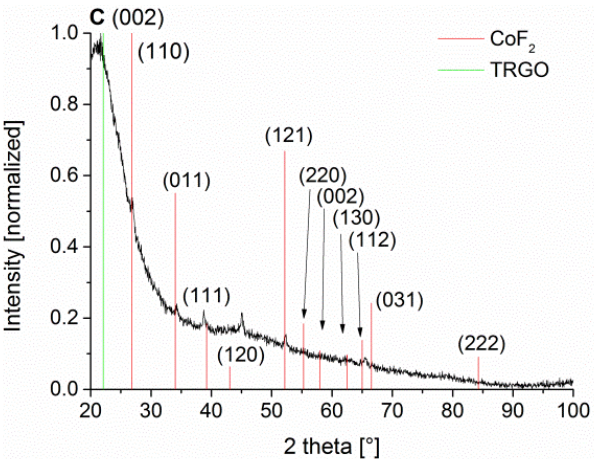

Figure S17: TEM and size distribution (top) PXRD (bottom. CoF_2–reference reflections in red from COD 9009073), of 0.5 wt % CoF_2–NPs in [BMIm][BF_4] @TRGO–SH from Co(AMD)$_2$.

PrF$_3$@TRGO-SH

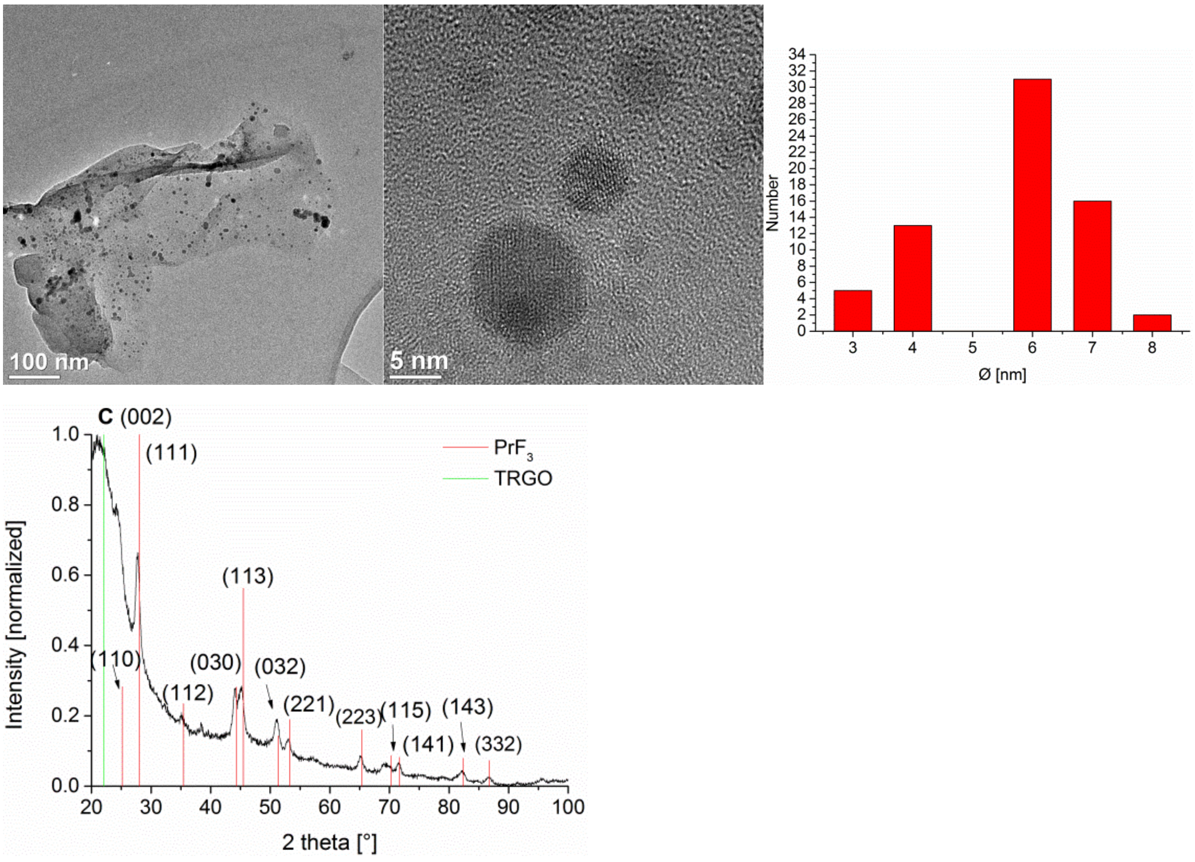

Figure S18: TEM and size distribution (top), PXRD and EDX (bottom, PrF_3-reference reflections in red from COD 1010984), of 0.5 wt % PrF_3-NPs in [BMIm][BF_4] @TRGO–SH from Pr(AMD)$_3$.

EuF$_3$@TRGO-SH

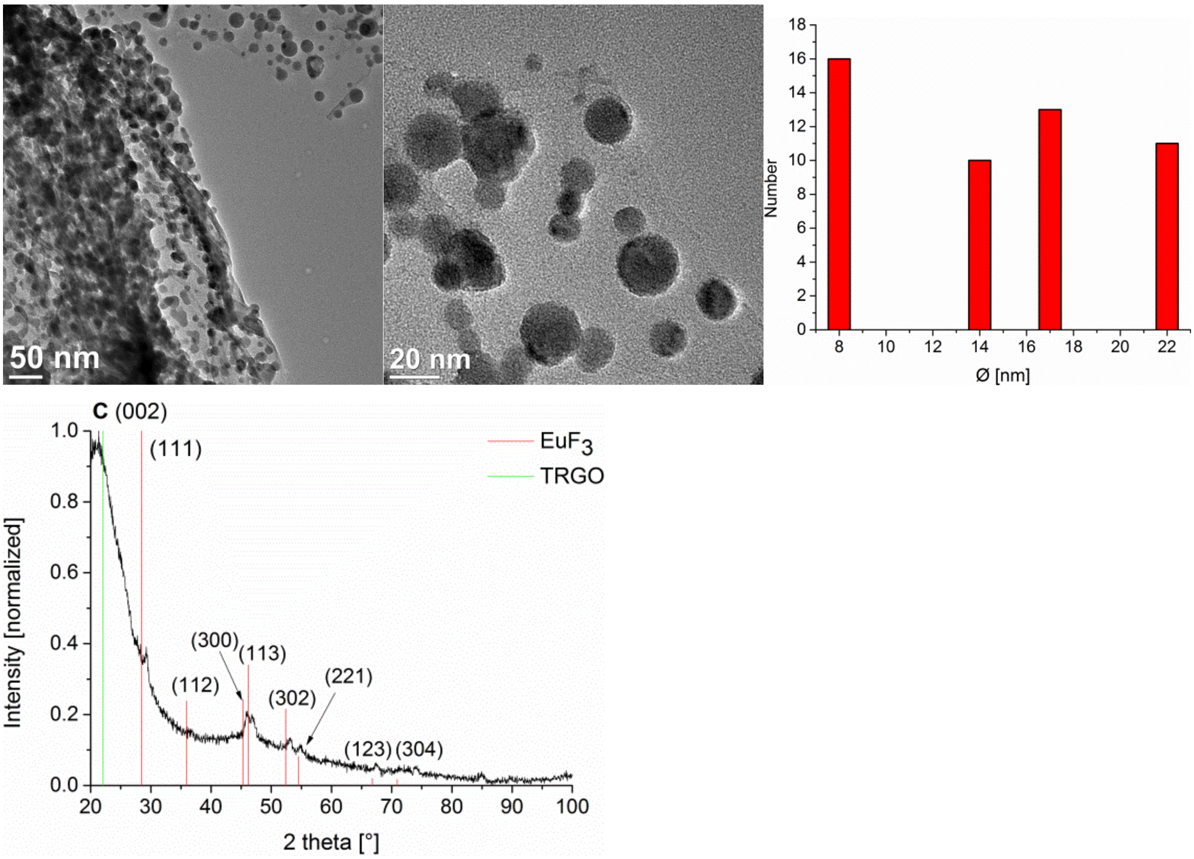

Figure S19: TEM and size distribution (top), PXRD and EDX (bottom, EuF_3-reference reflections in red from ICDD 33–0373), of 0.5 wt % EuF_3-NPs in [BMIm][BF_4] @TRGO–SH from Eu(dpm)$_3$.

References

1. Marquardt D.; Vollmer C.; Thomann R.; Steurer P.; Mülhaupt R.; Redel E.; Janiak C. *Carbon*, **2011**, 49, *1326-1332.*
2. Marquardt D.; Beckert F.; Pennetreau F.; Tölle F.; Mülhaupt R.; Riant O.; Hermans S.; Barthel J.; Janiak C. *Carbon*, **2014**, 66, 285-294.
3. Thermo Scientific XPS, http://xpssimplified.com/periodictable.php, (accessed December 2016).
4. Moulder J. F.; Stickle W. F.; Sobol P. E.; Bomben K. D. *Handbook of X–ray Photoelectron Spectroscopy*, Chastain J.; Perkin–Elmer Corporation, Minnesota, 1992.
